# Implementation Determinants of Problem-Solving Therapy Delivered by Near-Peer Lay Counselors for Youth Living with HIV in Botswana: Lay Counsellor Perspectives

**DOI:** 10.1007/s43477-024-00126-6

**Published:** 2024-07-15

**Authors:** Charisse V. Ahmed, Amelia E. Van Pelt, Alison M. Buttenheim, Ohemaa Poku, Bridgette M. Rice, Elizabeth D. Lowenthal, Merrian J. Brooks

**Affiliations:** 1 David Gefen School of Medicine, University of California, Los Angeles, USA; 2 University of Pennsylvania School of Nursing, Philadelphia, USA; 3 Feinberg School of Medicine, Department of Medical Social Sciences, Northwestern University, Chicago, USA; 4 Department of Psychiatry, University of Pennsylvania Perelman School of Medicine, Philadelphia, USA; 5 HIV Center for Clinical, Behavioral Studies at the New York State Psychiatric Institute and Columbia University, New York, NY, USA; 6 Villanova University, Fitzpatrick College of Nursing, M. Louise, Villanova, USA; 7 Departments of Pediatrics and Biostatistics, Epidemiology and Informatics, University of Pennsylvania Perelman School of Medicine, Philadelphia, USA; 8 Departments of Pediatrics, University of Pennsylvania Perelman School of Medicine, Philadelphia, USA; 9 Children’s Hospital of Philadelphia Craig Dalsimer Division of Adolescent Medicine, Philadelphia, USA

**Keywords:** Adolescents, HIV, Botswana, Mental health, Implementation science, Friendship bench

## Abstract

An evidence-based psychological intervention, known as Friendship Bench, for depression and anxiety was adapted among adolescents living with HIV (ALHIV) in Gaborone, Botswana, and renamed Safe Haven. The purpose of this study was to qualitatively describe the barriers and facilitators that influence the implementation of Safe Haven from the perspective of peer counselors delivering the intervention in Gaborone, Botswana. We conducted a secondary analysis using qualitative data from a pilot study to evaluate Safe Haven. Eight peer counselors participated in semi-structured interviews to describe their experiences with implementing Safe Haven during the pilot. We analyzed the interview data thematically using the Consolidated Framework for Implementation Research to guide theme development. We identified six barriers and two facilitators of Safe Haven implementation. The barriers were 1) client reticence and confidentiality concerns, 2) parent disapproval, 3) client accessibility, 4) counselor psychological wellbeing, 5) scheduling conflicts 6) limited financial resources for counselors. The facilitators were 1) peer delivery of counseling was deemed more acceptable among adolescents than counseling delivered by older adults, and 2) the counselors placed high value on the intervention. We found that parental disapproval and shared trauma among counselors and clients are key barriers that may negatively impact implementation outcomes such as sustainability and penetration. To improve accessibility of the intervention, peer counselors recommended implementation in school settings and to educate parents on mental health. Overall, the barriers and facilitators identified in our study can guide larger scale implementation of Safe Haven among ALHIV in resource-poor settings.

## Introduction

Mental illness is a significant contributor of disease burden and mortality among adolescents worldwide (Kieling et al., 2011; Mokdad et al., 2016; Shorey et al., 2021; Stupar et al., 2021; Yatham et al., 2018). Approximately 25% of adolescents aged 10 to 19 years, globally, are at risk for clinical depression, with one of the highest risks occurring among adolescents in Africa (Shorey et al., 2021). The burden of mental illness is of particular concern among adolescents living with HIV (ALHIV) (Kemigisha et al., 2019; Kim et al., 2014; Malee et al., 2011; Olashore et al., 2021; Vreeman et al., 2017). In sub-Saharan Africa, the prevalence of depression, anxiety, and other psychiatric disorders among ALHIV has been reported to be between 20 and 27% (Olashore et al., 2021). Particularly in Botswana, depression and suicidality have been reported as reasons for non-adherence among ALHIV (Yang et al., 2018). Additionally, 17% of Botswana ALHIV are at risk for a mental health disorder and therefore need additional screening (Lowenthal et al., 2012).

Botswana has an HIV prevalence of about 21% with a third of new infections occurring among adolescents and young adults aged 15 to 24 years (UNAIDS, 2020). Botswana also has fewer than 2 psychologists, psychiatrists, and social workers per 100,000 people (World Health Organization, 2011). To fill this gap, the Safe Haven intervention was piloted in Botswana to meet the mental health needs of ALHIV. Safe Haven is an adaptation of the Friendship Bench intervention that was first introduced in Zimbabwe. The Friendship Bench is an evidence-based mental health intervention primarily centered around problem-solving therapy delivered by lay health workers largely within primary care settings in low- and middle-income countries (LMICs) (Abas et al., 2016; Chibanda et al., 2011, 2016). PST is considered effective at reducing depression and anxiety symptoms across multiple settings and contexts, including in adolescent populations (Bell & D’Zurilla, 2009; Eskin et al., 2008; Pierce, 2012; Zhang et al., 2018). Friendship Bench shows promise among people living with HIV; the intervention was considered feasible and acceptable to implement among 32 Zimbabwean adults with poor antiretroviral therapy (ART) adherence and at least mild depression (Abas et al., 2018). The original Friendship Bench model was implemented by lay health workers who were older adult women known as “grandmothers” (Chibanda et al., 2011).

The adaptation of Friendship Bench into the Safe Haven intervention involved the use of near-peer lay counselors in Botswana. A near-peer lay counselor is a counselor of similar age to those they are counseling. Since the Safe Haven intervention targeted adolescents aged 12 to 25 years, the near-peers were young adults up to age 30. Table 1 highlights the intervention components of Safe Haven. The process of Friendship Bench adaptation into Safe Haven involved using methods informed by community-based participatory research (Brooks et al., 2021). The original components of the Friendship Bench intervention are described elsewhere (Chibanda et al., 2011). Safe Haven lay counselors were directly supervised by a clinical team of physicians and mental health professionals, and the support structure comprised post-counseling debrief and support group sessions for counselors. In the debrief sessions, lay counselors received group support led by a mental health professional. The purpose of the debrief sessions was to help lay counselors navigate challenging cases from their counseling sessions. In the support group sessions, the counselors received psychological support through group counseling led by a psychologist. Counselors also had the option to receive individual counseling if needed.

Implementation science offers a systematic approach regarding contextual factors to consider when integrating evidence-based interventions into practice. Currently, there is a paucity of implementation research targeting evidence-based mental health interventions among ALHIV in sub-Saharan Africa. Therefore, the purpose of this study was to qualitatively describe implementation determinants (i.e., barriers and facilitators that influence implementation) of Safe Haven from the perspective of near-peer counselors delivering the intervention to ALHIV in Gaborone, Botswana. This study will aid in the preparation of a larger scale implementation of Safe Haven among adolescent populations in resource-poor settings and inform the development of strategies to enhance implementation of this evidence-based intervention.

## Methods

### Study Design and Sample

This qualitative descriptive study is a secondary data analysis of interview data collected from eight of the nine near-peer lay counselors who participated in the Safe Haven intervention pilot in Gaborone Botswana from 2018 to 2019. One of the nine original counselors was not interviewed due to dropout. The counselors participated in semi-structured interviews in November 2019 and in November 2020, using the same interview questions on both occasions. Given that the November 2020 interviews yielded more in-depth information due to increased probing, only the data from the November 2020 interviews were utilized in our analysis. The interviews were designed to understand immediate needs for adaptation in preparation for a larger rollout of the pilot intervention. The counselors were asked questions like “Can you tell me about your experience working with youth that needed your assistance?”, “Were there any topics you felt were hard to talk with the youth about?”, and “What other thoughts or recommendations do you have to others who would be in your position one day?” Research assistants were fluent in English and Setswana and language use during the interviewers was determined by participant preference. Sometimes the counselors used both English and Setswana. All interview transcripts were transcribed in the language used during the interview and subsequently translated to ensure they were entirely in English. The English transcripts from the eight counselor interviews were used for qualitative analysis. The eight transcripts derived from the interviews provided rich data with a median word count of 7,595.

### Study Setting

The near-peer lay counselors were youth^1^ in between the ages of 21 and 28 (median age was 24), and all but one counselor reported living with HIV their whole life. Adolescent clients were screened for depression and anxiety symptoms during routine HIV care visits at the Botswana-Baylor Children’s Clinical Center of Excellence (BBCCCOE) in Gaborone, Botswana, and those with mild and moderate symptoms were recruited for the pilot study. The clients were able to participate if they screened positive for mild to moderate depression on the Shona Symptom Questionnaire (SSQ) or Patient Health Questionnaire-9 (PHQ-9) or screened positive for mild to moderate anxiety on the Generalized Anxiety Disorder 7 (GAD-7) scale. The age ranges of the clients were 13 to 24 years, and all clients were living with HIV.

The lay counselors were implementing Safe Haven with clients recruited from the BBCCCOE which is a government facility that is supported by Baylor College of Medicine with some personnel support and technical expertise. Clients are pediatric and young adult patients aged 0 to 26 years who are living with HIV. There are on average 40 patients seen per day. The near-peer lay counselors who participated in the pilot were not health care providers in the BBCCCOE clinic; rather, they were hired to implement the pilot study at the clinic. There is one psychologist, one social worker, three nurses, and up to five HIV clinicians working at the clinic.

To maintain confidentiality in the reporting of our results, each counselor was given a pseudonym. The following pseudonyms were used for the counselors who were men: Obonye, Tebogo, Mpho, Oratile, and Neo. The pseudonyms used for the counselors who were women were Lorato, Keeya, and Gorata. All repetitive words and natural pauses were removed from the quotes taken from the interviews to improve textual clarity. Lastly, we used Consolidated criteria for reporting qualitative studies (COREQ) checklist (Tong et al., 2007) to provide explicit reporting of our research team, study design, analysis, and findings (see Supplemental Material File 2 for COREQ checklist).

### Data Analysis

**Fig. 1:**
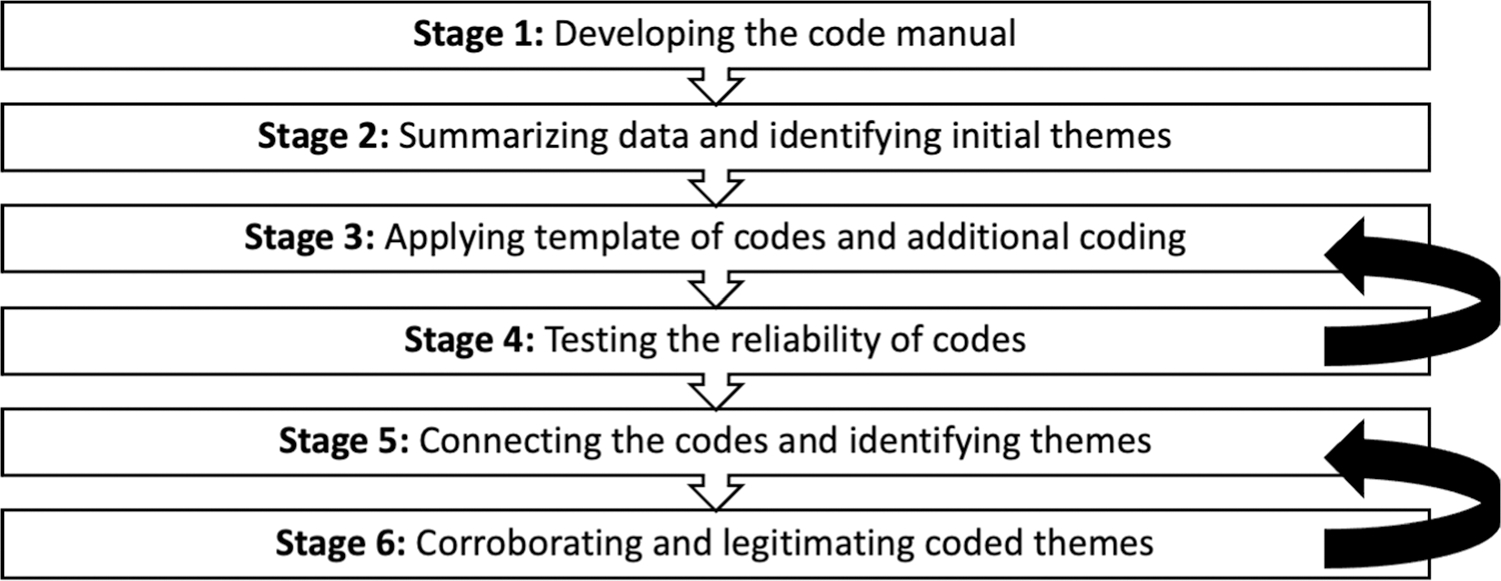
Modified Fereday and Muir-Cochrane (2006) hybrid thematic analysis approach

Fereday and Muir-Cochrane’s (2006) inductive and deductive (or hybrid) approach to qualitative thematic analysis was utilized to analyze data derived from the interview transcripts. The epistemological viewpoint typically underpinning thematic analysis is constructivism which aims to understand a phenomenon based on subjective views of the participants (Vaismoradi et al., 2013). Feredey and Muir-Cochrane (2006) describe six stages that involve coding data, categorizing codes, and identifying themes. The six stages to the hybrid approach are visually outlined in Fig. 1. In Stage 1, the first author (C.V.A) developed a codebook by deductively generating a priori codes (i.e., generated prior to empirical review of the data) as guided by all of the domains and constructs from Damschroder and colleagues’ original Consolidated Framework for Implementation Research (CFIR) (Damschroder et al., 2009). After a thorough review of the eight transcripts, C.V.A created an initial codebook with a priori and preliminary a posteriori codes (i.e., generated after a post-review of the data). In Stage 2, initial themes (or patterns) were identified by summarizing the data based on participant responses to the questions from the semi-structured interviews. During this stage, the second author (A.V.P) read two of the transcripts to capture initial themes which were used to refine the codebook. Lastly, member checking among the near-peer counselors that were interviewed was used to validate the initial themes and further refine the codebook. Member checking, also known as participant or respondent validation, is a technique used by researchers to determine the credibility of qualitative research findings (Birt et al., 2016). Member checking of initial themes was utilized to ensure that the counselors’ perspectives were not misrepresented. Initial themes with supporting anonymous quotes from the transcripts were presented to the counselors in a virtual group meeting where they confirmed the accuracy of the themes; their insights were also used to finalize the codebook.

In Stage 3, C.V.A. coded all eight interviews using NVivo (QSR International Pty Ltd, 2020). In Feredey and Muir-Cochrane’s (2006) original framework, Stage 4 (i.e., testing the reliability of the codes) was initially Stage 2, which only allowed for testing the reliability of the a priori codes. However, the modified framework outlined in Fig. 1 allowed for intercoder reliability testing to occur across a priori and a posteriori codes. To establish intercoder reliability, Stage 4 involved a second coder (A.V.P) who coded half of the interview transcripts using the final codebook. Discrepancies in coding were addressed until the first and second coder reached a percentage agreement of at least 95% (O’Connor & Joffe, 2020). In Stage 5, C.V.A identified themes by using a matrix to cluster salient barriers and facilitators by CFIR domains; barriers and facilitators were considered themes (See supplementary material).

In Stage 6, C.V.A corroborated the themes by discussing each barrier and facilitator with A.V.P to collectively refine themes by CFIR domain. The collective refinement of themes initially resulted in 17 determinants. The themes were later synthesized and condensed to eight determinants based on the most salient themes per CFIR domain with guidance from the senior author (M.J.B) who worked closely with the counselors to implement Safe Haven (see Supplemental Material File 1 for theme development process).

### Ethical Clearance

The near-peer lay counselors signed a written informed consent form approved by the Institutional Review Boards at the Health Research and Development Committee (HRDC) in Botswana, the University of Pennsylvania, and the Children’s Hospital of Philadelphia.

## Results

Eight implementation determinants were identified, six that were barriers and two that were facilitators. The barriers identified were: 1) client reticence and confidentiality concerns, 2) parent disapproval, 3) client accessibility, 4) counselor psychological wellbeing, 5) scheduling procedures, and 6) lack of financial resources. The facilitators were: 1) peer delivery of counseling and 2) counselor perceived value of the intervention. Table 2 displays the barriers and facilitators with their relevant CFIR domains and constructs. Although there are more barriers than facilitators presented, the counselors suggested or have successfully used strategies to address some of the barriers. Where solutions were suggested, these are discussed along with the barriers below.

### Barriers

#### Client Reticence and Confidentiality Concerns

All but one of the peer counselors revealed that clients are usually reticent or reluctant to confide in them during initial sessions. According to Neo, adolescents may fear that the counselors will disclose information shared during the counseling sessions with the doctors at the clinic. Two counselors, Tebogo and Mpho, suggested that clients may not feel comfortable confiding in counselors whom they already knew or have seen prior to the counseling sessions. Tebogo said, “The first session that’s where I get problems where I have to make the client to feel comfortable when talking to you because some of these clients [they] get to see you so trying to talk to them when they had seen you before it’s a barrier… I have seen [one client] around because we live in the same neighborhood.” Similarly, Mpho stated, “In most cases it’s not every youth or everyone that’s free to talk to someone their age… so most of the time… the clients that we see here know us… Maybe it’s just the human mind… if I tell you something personal and I see you in public… automatically I will start thinking maybe you told someone or you are going to just say what I told you out and everyone hears it.”

Despite initial concerns regarding confidentiality and privacy, counselors agreed that client trust usually improves as the sessions progress: “…the first session I should think is the most difficult because this person doesn’t know whether to trust you or not because of how fast the information can travel nowadays. So, it’s a very difficult thing but as time goes, as the client [begins] to trust you, it’s fine. The problem is when you start the session” (Atujuna et al.). Some counselors explained how they resolved this challenge by ensuring confidentiality and utilizing communication strategies to gain trust and establish rapport among their clients. For instance, Gorata stated, “The issue of them not opening up was for [me] to set up a platform where I can make them feel free that this is just me and you. [I] get them to tell [me] about themselves, what they are doing, school, what they like doing, hobbies… to make them open up… we just had to do icebreakers.” However, Oratile explained how he had difficulty engaging a client who was reticent throughout the six sessions: “I once had a client who never really said anything… [he] wasn’t really opening up across all repeated sessions and we then ended sessions just like that, we didn’t cover much though he had a few list of things… there were like four or five problems and then some of them we didn’t go over them… So we only attended like two… we should be glad as a counselor that at least you have managed to cover other problems because maybe [there is] one problem [that] needs two sessions or three sessions.” Oratile recommended an increase from six sessions to twelve, when necessary, to accommodate clients who may take longer to open up or have problems that take longer than six sessions to address.

Among her reticent clients, Lorato would foster rapport building by saying “let’s just sit down and pretend like you and I are friends.” Although most counselors were eventually able to develop camaraderie among their clients, they also had to reinforce professional boundaries: “But most of my clients… they left here very happy, to the point in whereby even after the whole sessions were done, even when they see me in the streets they will be like ‘Oh! Wow! I wish I could see you again, I wish we could have your contacts or something like that.’ I am not allowed to do that so I was like ‘you could just keep see[ing] me here [at the facility] or whatever’” (Maphisa et al.). Likewise, Neo said “this one client wanted my number, I was really saddened by the fact that we were not supposed to give clients our numbers because like he felt like I was sort of like a big brother and we [had a] fraternal type of like relationship or like he was always looking forward to coming in.”

#### Parent Disapproval

Parent disapproval of Safe Haven may impact client participation in the intervention. Parents may not be willing to accept the intervention because they “don’t intend to understand the importance of counseling” (Keeya). Another issue is that parents may have discordant attitudes about the intervention: “Before [the clients] come for counseling, one needs parental consent. So, the other parent will agree and the other one will not agree. So, while I want to be counseled you know it’s going to be a conflict between [the] parents now” (Keeya). According to Gorata, some parents interfere with the counseling sessions or do not allow their children to participate after the client discloses information shared during the sessions: “…the challenge that we encountered most was that parents want to know what you were talking about. You see, that was a problem. So, the big challenge was that after [the clients] told them, then it was like the parents told them don’t go there again.”

Parental nonacceptance of Safe Haven was tied to cultural norms and societal views about counseling. Obonye highlighted beliefs about counseling held by older adults: “Since us we grew up in a society where we don’t usually share our feelings, well our parents are not those kind of people [who] believe a child can be depressed, can have anxiety, can have [these] other problems because they think they are for older people only… they don’t believe the youth can have those problems.” Neo’s comment regarding cultural views around counseling may also explain parental nonacceptance of the intervention: “when it comes to a lot of [Batswana] families there is this feeling towards psychology that’s there, you see? Where they feel like ‘Ah, I don’t want to go see a shrink’ or ‘I am too good for psychology’ or ‘psychology is only for people that [have] something wrong with them,’ you see?”.

As suggested by Obonye, parental interference may also explain the reasons why clients are reluctant to confide in the counselors about serious issues such as domestic violence: “normally in our society… when a kid [is] going to see a counselor, to our parents it’s like we are going to reveal family secrets… let’s say maybe the child is being bullied at home, is being abused and what, that process of them going to see a counselor even the parents will be on edge that this child may expose us. So that kind of environment will cause that child to not say anything willingly. They just avoid most of the questions and answer them with short-ended questions to avoid revealing more information.” Obonye tried to resolve this issue by educating the parents: “Basically we try to talk to the parents… we tried to show them what we really do. It’s not about getting to know their secrets. We just show them that all we want to know is to understand what the child is going through… not what’s going on at home.”

#### Client Accessibility

Since Safe Haven was implemented in a clinic where the clients received their HIV care (i.e., BBCCCOE in Gabarone), the counselors made comments regarding ways in which this intervention may not be accessible if expanded to other adolescent populations. For instance, Oratile stated that the intervention is not accessible to adolescents with disabilities: “we have disabled people… it’s our wish to attend [to] them because they also need therapy sessions but [the] disabled people cannot come here because some of them need special transport to be here.” Additionally, the location of the counseling sessions did not seem ideal for clients who lived far away or in rural areas. Keeya said, “even the rural areas they need counseling… the kids there they need counseling, they deserve it also… they don’t get a chance to see something like [Safe Haven].” According to Oratile, “the only hiccup [the clients] have is transport because some come as far as Mochudi, Ramotswa, Tlokweng [villages up to an hour away with direct transportation].”

Counselors also reported that time acted as a barrier to accessing the intervention because the sessions conflicted with school hours. Keeya and Oratile suggested implementation in school settings to address this barrier. Oratile said, “why can’t [we] go to school and attend [to] them during lunch time or during their part time of the study time, so that then we avoid them coming here and also going back… and when the client comes you have to report to the parent that the child has arrived… with us going to schools it will cut the whole barrier because a parent can call saying the child has not arrived on time… if we could go to schools and meet them in schools and come back, [then] they don’t have to miss school and stuff like that or missing school it affects them also academically.”

#### Scheduling Procedures

Several counselors implied that the initial procedures used to schedule clients for counseling sessions were barriers to Safe Haven implementation. At the beginning of the pilot, when counselors were scheduling their clients by appointment only, clients were missing their scheduled appointments or attending late: “The challenge is always like coming here and also rescheduling issues… because maybe I will come here knowing my client will be here at two o’clock and then at half past two they will be telling me ‘no, I have family issues’… and then we reschedule to a different date [to] accommodate my client with me now, and I have to make sacrifices for my client because I understand it’s one of the things that we were told about. That sometimes you are going to have difficulties that you are going to invade some of the personal, private time” (Oratile). Similarly, Tebogo said “if we decided that we should meet with the client around one, then the client decides to come around two… it’s a problem because I do have other commitments outside there. So, waiting an hour for a client is a problem.” Scheduling can also be problematic for counselors who are in school or who have other jobs: “[scheduling] was one of the most troubling things because I’m a tertiary student… because I have to like fix both of my schedules looking at what I have at school because it’s my first priority” (Oratile).

The block system appears to be a plausible solution to the scheduling issue. According to Tebogo, “they [the research team] have come up with a system of blocks where you are given four hours period to wait for a client, then the next four hours they bring another counselor… the scheduling was okay after they introduced the blocks.” Additionally, Obonye suggested to address the scheduling issues by working full-time to accommodate more clients throughout the week: “I think the solution that we need is basically to have [our] own space where we will be there full time to assist whoever comes whether we know him, whether we don’t know her. Just what ever help they need at a specific time they will know where to find us.”

#### Lack of Financial Resources

Several counselors emphasized the need for basic financial support, particularly more transport funding to carry out their job responsibilities. Three counselors expressed that their current pay was not enough to afford transportation to the location of the counseling sessions, especially with recent fare increases. Others also mentioned the need for money to meet their personal needs with statements like “I am not financially stable” (Atujuna et al.) and “the money that we get is not even enough to support our own selves” (Obonye). Tebogo even expressed that he felt underpaid for his time: “I’ve invested so much in this program but the rewards are not necessarily meeting my expectations.” Gorata highlighted the need to receive pay that is commensurate with her new skillset: “My wish is for this thing to not just be a volunteer thing for us. I mean we are the first people in Botswana to having started this. And I am sure in the future we are going to have more people being a part of this, right? So, my wish is that we should not be seen just as volunteers… We may not be qualified like degree, diploma kind of qualified, but certificate yes, we have experience of a year by now. We have an experience of a year, and at some point in the future they should consider it for us it’s a job.” The lack of financial resources for transportation was also related to the issues of sporadic attendance among the clients: “money is a problem… sometimes [the clients] will just pop up out from nowhere… Imagine if I get a call like now to come here… it’s gonna take me a long time, it’s gonna need money also. What if at that certain time I don’t have any money with me? You see that’s the kind of problem that we face” (Obonye).

#### Counselor Psychological Wellbeing

Counselors commonly reported challenges with experiencing the same hardships as their clients which can compromise their ability to provide problem-solving therapy. One counselor shared that his clients would “say something that will remind [him] of [his] own problems” (Stockton et al.). Another detailed how such discussions can “open up some old wounds [and] bring some flashbacks” (Obonye). Counselors mentioned ways in which they tried to conceal their emotions during the counseling sessions such as “I can make an excuse to go drink water” (Oratile) and “I had to pretend like I wasn’t going through what [the client] is going through” (Maphisa et al.). These emotionally triggering experiences can make it difficult for counselors to continue with the therapy sessions and may therefore prompt an unwanted referral to another counselor. For instance, Obonye said “there are scenarios where my client will be talking about [a] problem that I am basically going through right now. But when I try to refer him to another counselor, he doesn’t want that other counselor. He wants specifically to see me.” Although counselors took advantage of the intervention’s psychological support structure to manage these stressors, counselors may feel morally obligated to continue providing counseling despite the emotional burden. Neo said, “when you have cases that hit close to home, sometimes it can happen that you [are] the best person to help the client… that’s the only reason why I didn’t refer it… I try my best to still be objective [and] help the clients find solutions.”

### Facilitators

#### Peer Delivery of Counseling

Chief among the facilitators was the high level of acceptability of peer-delivery of counseling. Peer counselors considered Safe Haven a better alternative to conventional counseling led by older adults because the delivery of counseling by peers eliminates communication barriers related to older age. Oratile compared the benefits of peer-delivered counseling to the counseling he received in junior secondary school: “in junior schools [the counselors] are older and [with peer counselors] there is no language barrier because [we] are both youths so they could say whatever they need to say but then it is easy for me to understand… [if] they use street language, I can also like get the message from that rather than someone who is older.” Oratile also believed that he was more relatable to his clients than his older adult counterparts: “[the clients] are younger than me but then they can open up and see he’s not really that old.” Two additional counselors made statements to reflect their relatability such as “even if I am old, I went to their level” (Gorata) and “we take ourselves as one as the youth [and] they see us as one and the same, people on the same level” (Obonye). Gorata stated that “[she] didn’t [act] like old ladies, like old Tswana parents [who] are very judgmental when [they] hear what [the clients] have to say.”

Counselors also emphasized that their similar age offers them the ability to talk about topics relevant to adolescents such as sex, relationships, social media, and cyber bullying. Oratile said, “[peer counselors] can talk about Facebook because [they] know Facebook, [they] can talk about Instagram because [they] know Instagram [but the older psychologist] doesn’t know Instagram.” Two counselors implied that they were able to talk to their clients about topics related to sex and relationships due to their age. Obonye stated, “we can talk for hours about literally anything [such as] relationships [and] sex lives, [as] long as it’s [just] us youth [I] don’t believe there is anything difficult to talk about.” On the other hand, Oratile implied that conversations around sex are difficult to talk about among older adults: “it’s much easier to work on all issues [and] even issues of sex… it’s easier to talk about them when I am with [the clients] because they open up… you know in our culture era we can’t talk to an elder about sex issues.” Oratile also stated his qualms about counseling provided by older adults: “when I was at junior school [I] knew that the only way I could get counseling [is] if I go to the society counseling people or I go to school [which is] totally uncomfortable for me because I will be going there with a relationship issue [and] I am not going to open up to someone older than me.”

#### Counselor Perceived Value of the Intervention

Counselors’ perceived value of the intervention may determine how motivated they are to implement problem-solving therapy within Safe Haven. Peer counselors were committed and personally invested in the implementation of Safe Haven because they acknowledge the positive impact that the intervention has on their clients. For instance, Mpho stated “the best part is [that] we can see the difference between how you communicate with the client… you see that they are now free… like you are really doing something to help someone.” Similarly, Lorato mentioned that she noticed “a different version” of her clients after the sessions and believes she “can actually help somebody” because the clients are learning how to solve their own problems as a result of the therapy. Tebogo discussed the benefits of providing counseling among his peers: “it was very exciting to work with young people such as me because it also shows me that all those challenges I go through, it’s not only me. There is someone going through them and it’s also exciting because I get to help my peers.”

Despite concerns regarding low compensation, the interviews suggest that counselors perceive their participation in Safe Haven as invaluable. Tebogo implied that his commitment to the intervention supersedes his desire for more compensation. He stated “I would still be here because I do believe in the program but [I] would hope in the long run things will change… I’ve invested a lot of time here and [that] time I could be using it to look for ways to be financially stable.” Tebogo also revealed that his compensation did not impact his participation or performance within Safe Haven: “I will not say [my pay] has derailed me from doing what I am doing here… it’s a personal choice to come here so… I should think it did not derail me.”

## Discussion

The implementation determinants we identified provide important insights for future implementation of Safe Haven and perhaps other lay-delivered psychological interventions targeting adolescents. We identified six main barriers to implementation of Safe Haven, including client reticence and confidentiality concerns, parent disapproval, client accessibility, counselor psychological wellbeing, scheduling conflicts, and limited financial resources for counselors. The two main facilitators we identified were 1) peer delivery of counseling was deemed more acceptable among adolescents than counseling delivered by older adults, and 2) the counselors placed high value on the intervention. Given the target population of Safe Haven, parent disapproval is a key barrier affecting implementation, and the counselors in our study offered potential solutions to this barrier.

Our study indicated that parental influences, an “Outer Setting” barrier (Table 2), are important factors to consider when implementing psychological interventions for adolescents. According to Damschroder and colleagues’ original CFIR framework, “Outer Setting” barriers refer to external influences on implementation of a given intervention or program (Damschroder et al., 2009). Although we did not identify an appropriate CFIR domain to map with our parent disapproval barrier (see Table 2), we classified parent disapproval as an “Outer Setting” barrier in light of the influence parents exert over their children’s capacity to utilize services. Parents are often the gatekeepers of mental health service utilization among adolescents across high-income countries and LMICs. Parental perceptions and stigmatization of counseling were identified as driving barriers to mental health service use among minority adolescents in the United States (Lu et al., 2021). As suggested by Neo in our study, MacCann and colleagues (2016) indicate that some sub-Saharan African parents view mental health counseling as stigmatizing and were raised to believe that mental health professionals are “shrinks”. In Wogrin et al.’s (2021) Friendship Bench model for ALHIV, peer counselors also faced resistance from caregivers, but found that pre-engagement activities helped to earn their approval. In our study, Tebogo recommended educating parents about counseling to remove myths or biases associated with the provision of counseling among their children, which can potentially be used as an implementation strategy in future iterations of Safe Haven.

The barriers identified in our study also provide several implications regarding the implementation outcomes of lay-delivered psychological interventions like Safe Haven. As identified in prior research (Adugna et al., 2020; Arzamarski et al., 2021), our findings suggest that “Outer Setting” (CFIR domain) barriers such as limited accessibility may impact recipient penetration. According to Proctor and colleagues (2011), recipient penetration is an implementation outcome that refers to the extent to which eligible individuals use a particular service. Our counselors recommended implementation of Safe Haven within school settings to improve client accessibility. Additionally, the block system was designed to address scheduling challenges by enabling the counselors to choose a four-hour period in which they were available to see clients, which specifically addresses a “Process” (CFIR domain) barrier. This strategy alleviated the burden of waiting for clients who missed their appointments and therefore may also improve recipient penetration.

In the context of the “Inner Setting” (CFIR Domain), our study highlights the importance of sufficient financial resources to promote sustainability as an implementation outcome. Lay health workers often have concerns regarding low compensation which may impact motivation to maintain the intervention (Ahmed et al., 2020; Wall et al., 2020). Similar research evaluating lay-delivered psychological interventions suggest that counselor motivations are important indicators of successful delivery and sustainability (Shahmalak et al., 2019; Verhey et al., 2020). As confirmed in a similar study (Verhey et al., 2020), we also found that lay counselors are motivated by personal gain, which represents “Characteristics of Individuals” (CFIR domain). Corroborating our findings, lay counselors of Friendship Bench and other psychological interventions often report counseling as a rewarding experience leading to personal growth such as improved self-confidence and altruism, and improved efficacy in managing their own problems (Shahmalak et al., 2019; Wallén et al., 2021). Counselor motivation may also improve implementation fidelity as motivated counselors are intentional about delivering the intervention correctly (Verhey et al., 2020).

Our findings also highlight potential advantages of utilizing peers to deliver psychotherapy. In the context of relative advantage (a construct within the CFIR domain “Intervention Characteristics”), we found that shared experiences and similar age make peer counselors more relatable among their clients than adult counselors. These advantages were corroborated in similar iterations of Friendship Bench adapted for adolescents (Broström et al., 2021; Wogrin et al., 2021). Our findings also indicate that peer counselors are more appropriate than adult counselors when engaging in discussions around sensitive topics with adolescent clients. In the Botswana context, our research is consistent with other research suggesting that adolescents would prefer to talk to their same- or similar-aged peers, rather than older adults, about sex and other sensitive topics (Delius & Glaser, 2002; Mutschler et al., 2021; Ntsayagae et al., 2008). Supporting our findings, Ahmed et al.’s study revealed that adult-aged lay health workers, known as expert clients, in Eswatini believed that they were not ideal candidates for discussions around sex with their adolescent clients due to their age (Ahmed et al., 2022). However, some adolescents may remain hesitant to engage in these discussions even amongst their adolescent peers. Wallén and colleagues found that adolescent clients participating in Friendship Bench in Zimbabwe were uncomfortable with discussing sex and relationships with peer counselors, and the peer counselors also felt inadequately prepared to address such topics (Wallén et al., 2021). Thus, future implementation of Safe Haven should ensure competencies around sex education and perhaps even incorporate sexual education within the counselor training.

Our findings also point to potential disadvantages of using peers to deliver psychotherapy, especially as it pertains to the individual characteristics of counselors and clients (CFIR domain “Characteristics of Individuals”). Wogrin and colleagues (2021) reveal that ALHIV who were clients within Friendship Bench had initial concerns around status disclosure even though their peer counselors shared their HIV status. As noted in similar studies (Broström et al., 2021; Shahmalak et al., 2019), we found that ensuring confidentiality can mitigate these concerns and that trust develops overtime. Contrary to Thoits’ findings regarding peer support (Thoits, 2021), peer counseling for adolescents may not be ideal when counselors share similar stressors as their clients. As found in other peer-delivered versions of Friendship Bench for adolescents (Wallén et al., 2021; Wogrin et al., 2021), our study indicates that peer counseling may involve shared trauma. While shared experiences can be an advantage in some contexts, shared trauma may put counselors at risk for post-traumatic stress, and may also increase self-disclosure and compromise professional boundary-setting (Mutschler et al., 2021; Tosone et al., 2012). Similar to our findings, Wogrin et al.’s (2021) Friendship Bench pilot revealed that peer counselors may feel obligated to take on the psychological burden of managing clients with complex needs. These factors may hinder successful peer counseling and lead to burnout and compassion fatigue, which may impact the sustainability of peer-based counseling. Therefore, future implementers should consider evaluating for shared trauma prior to pairing counselors with clients. Trauma assessments as well as screening for childhood adversity (e.g., Adverse Childhood Experiences-International Questionnaire) can be used among counselors and clients prior to initiating therapy sessions.

While our study findings corroborate the utility of CFIR to capture implementation determinants, there are gaps regarding its applicability across LMICs. Although new constructs were added to CFIR after our analysis (Damschroder et al., 2022), Means and colleagues (2020) provide specific constructs relevant to the LMIC context. One of the constructs proposed was community characteristics, a determinant which considers the sociocultural and religious context of the consumers of an intervention. As implicated by our research, cultural norms and views regarding counseling are essential determinants of successful implementation of Safe Haven. Additionally, Means and colleagues (2020) proposed a new domain, characteristics of systems, to capture factors within healthcare systems that may impact “Inner Setting” and “Outer Setting” constructs. For instance, resource source considers resources for entire healthcare systems such as domestic government resources, bilateral developmental aid, and private foundation support (Means et al., 2020). As suggested by our study, the lack of financial resources is a concern across several interventions implemented in LMICs, thereby warranting deeper consideration to resource source as a potential determinant of Safe Haven implementation.

### Policy Implications

Our findings regarding parental disapproval demonstrate the need to address policies that may hinder access to mental health services among adolescents in Botswana. According to Botswana’s primary mental health legislation, individuals under 16 years of age cannot apply for voluntary inpatient treatment without parental consent (Maphisa, 2019). Additionally, Botswana’s mental health laws offer limited protections for persons with mental disorders (Maphisa, 2019), which may further exacerbate access to psychological interventions among adolescents. Therefore, implementation strategies are needed to garner parental approval of adolescent engagement in psychological interventions and there remains a need for robust policy infrastructure to further support adolescents with mental health needs.

### Strengths and Limitations

A major strength of our study is our use of a systematic inductive-deductive approach to analyzing our interview data. Another strength was that we used rich transcript data as our unit of analysis and relied on two coders (i.e., C.V.A and A.V.P) to enhance inter-rater reliability. Regarding our limitations, our analysis was based on eight available transcripts, and we were not able to determine if more interviews were needed to achieve data saturation. However, our themes do provide some evidence of saturation through the redundancy of ideas represented by each counselor across the themes and based on themes found in other studies evaluating similar iterations of Friendship Bench implementation for adolescents (Broström et al., 2021; Wallén et al., 2021; Wogrin et al., 2021). Second, we were limited in our ability to apply all the constructs within the CFIR framework to our analysis given that the near-peer lay counselor interview guide did not originally account for any of the CFIR domains and constructs. Lastly, our determinants were derived from the perspectives of peer counselors only. However, client views regarding the Safe Haven intervention were reported elsewhere (Garriott et al., 2023). Parental views regarding Safe Haven are warranted to corroborate the findings from this study. Interviewing parents may provide additional insights into ways in which the intervention can be further modified to promote acceptability and recipient penetration. These limitations all point to the need for additional implementation and effectiveness studies of adapted Friendship Bench interventions to further characterize determinants of success. For example, current research in progress is evaluating determinants and developing implementation strategies to optimize Friendship Bench implementation (Verhey et al., 2021).

## Conclusion

We identified eight determinants which may facilitate or hinder successful implementation of Safe Haven, a brief, evidence-based psychological intervention tailored for ALHIV in Botswana. The determinants highlight the advantages and disadvantages of utilizing peers to successfully deliver psychotherapy. Our findings also highlight the utility and limitations of CFIR for identifying implementation determinants in LMICs. Overall, our research can guide future implementation research evaluating the determinants of Friendship Bench tailored for adolescents across resource-poor settings. Our research also aligns with current efforts to leverage implementation research to address adolescent mental health disparities particularly in LMICs. Future research is needed to corroborate our findings through parent perspectives, to develop and test implementation strategies to address the barriers identified in our study, and to ultimately meet the needs of peer/near-peer counselors and their adolescent clients.

## Supplementary Material

Ahmed_Implementation Determinants of Problem-Solving Therapy S1 (COREQ Checklist)

Ahmed_Implementation Determinants of Problem-Solving Therapy S2 (Theme Development Matrix)

## Figures and Tables

**Table 1:** Depiction of friendship bench adaptation in botswana

Safe haven intervention components

• Six problem-solving therapy sessions delivered by near-peer lay counselors
• Protocolized problem-solving therapy training for near-peer lay counselors
• Clinical supervision of near-peer lay counselors by physicians and mental health professionals
• Post-counseling debriefs for near-peer lay counselors

**Table 2:** Implementation determinants of the adapted safe haven and related CFIR domains and constructs

Barriers	CFIR domain	CFIR construct

*Client reticence and confidentiality concerns*: During initial sessions, clients may be reluctant to confide in the counselors due to initial lack of trust or confidentiality concerns	Characteristics of individuals	Knowledge and Beliefs about the Intervention
*Parent disapproval*: Parents do not approve of client participation in friendship bench which may partially be explained by cultural views and stigmas around counseling	Outer setting	*
*Client accessibility*: The location for friendship bench implementation is not suitable for clients who have disabilities or live far, and for clients who need counseling during school hours	Outer setting	Patient needs and resources
*Counselor psychological wellbeing*: Counselors experience the same hardships as their clients which makes it difficult for them to continue with counseling sessions	Characteristics of individuals	Other personal attributes
*Scheduling procedures*: Scheduling by appointment is inconvenient for counselors because clients often attend their appointments late or miss their appointments completely	Process	Executing
*Lack of financial resources*: Counselors have limited financial resources to carry out their work and meet their personal needs	Inner setting	Available resources
Facilitators		
*Peer delivery of counseling*: Friendship bench implementation by peer counselors is more advantageous than conventional counseling provided by older adults due to age-related cultural similarities between the peer counselors and clients	Intervention characteristics	Relative advantage
*Counselor perceived value of the intervention*: Counselors value friendship bench because they understand the benefits of their participation for their clients and for themselves	Characteristics of individuals	Knowledge and beliefs about the intervention

*Note.* We did not identify a suitable construct from Damschroder and colleagues’ original CFIR to align with the “Parent Disapproval” theme

## Data Availability

The datasets are available from the corresponding authors on reasonable request.

## Footnotes

1 In Botswana, youth are considered any person between the ages of 12 and 35 years Lesetedi, G. (2018). High youth unemployment in Botswana: A case of policy failure or poor research. *Mosenodi: Botswana Journal of Educational Research and Social Sciences, 21 (1): 127, 136*. Ibid.
