# Supplementary material for: Implementation Determinants of Problem-Solving Therapy Delivered by Near-Peer Lay Counselors for Youth Living with HIV in Botswana: Lay Counsellor Perspectives
Source: Glob Implement Res Appl. Author manuscript; Available in PMC 2025 Dec 1. (PMC11905926; doi:10.1007/s43477-024-00126-6)
Supplement: Ahmed_Implementation Determinants of Problem-Solving Therapy S1 (COREQ Checklist) [file NIHMS2051337-supplement-Ahmed_Implementation_Determinants_of_Problem-Solving_Therapy_S1__COREQ_Checklist_.docx]

**Consolidated criteria for reporting qualitative research (COREQ) Checklist**

| **Domain 1: Research team and reflexivity** | | | |
| --- | --- | --- | --- |
| **Personal Characteristics** | | | |
| 1 | Interviewer/facilitator | Which author/s conducted the interview or focus group? | Merrian Brooks (senior author) and research assistants (Patlo Entaile, Kealeboga Morapedi, Makhetha Monyane-Pheko) |
| 2 | Credentials | What were the researcher's credentials? E.g. PhD, MD | Researchers: PhD, MD, MS |
| 3 | Occupation | What was their occupation at the time of the study? | Physician, Graduate Student, Postdoctoral Fellow (epidemiology background) |
| 4 | Gender | Was the researcher male or female? | Females |
| 5 | Experience and training | What experience or training did the researcher have? | Experience in qualitative research, semi-structured interviews, master’s level training in research methods. |
| **Relationship with participants** | | | |
| 6 | Relationship established | Was a relationship established prior to study commencement? | Yes |
| 7 | Participant knowledge of the interviewer | What did the participants know about the researcher? e.g. personal goals, reasons for doing the research | The consent forms stated that the researchers were interested in learning about the lay counselors’ experiences with the Safe Haven intervention so that the intervention can be improved or changed, if needed. |
| 8 | Interviewer characteristics | What characteristics were reported about the interviewer/facilitator? e.g. Bias, assumptions, reasons and interests in the research topic | None except as above |
| **Domain 2: study design** | | | |
| **Theoretical framework** | | | |
| 9 | Methodological orientation and Theory | What methodological orientation was stated to underpin the study? e.g. grounded theory, discourse analysis, ethnography, phenomenology, content analysis | Hybrid inductive/deductive analysis |
| **Participant selection** | | | |
| 10 | Sampling | How were participants selected? e.g. purposive, convenience, consecutive, snowball | Purposive (based on participants of the pilot) |
| 11 | Method of approach | How were participants approached? e.g. face-to-face, telephone, mail, email | Face-to-face |
| 12 | Sample size | How many participants were in the study? | Not applicable |
| 13 | Non-participation | How many people refused to participate or dropped out? Reasons? | Not applicable |
| **Setting** | | | |
| 14 | Setting of data collection | Where was the data collected? e.g. home, clinic, workplace | Clinic private rooms |
| 15 | Presence of non-participants | Was anyone else present besides the participants and researchers? | No |
| 16 | Description of sample | What are the important characteristics of the sample? e.g. demographic data, date | The 8 participants were in between the age of 21 to 28; 5 participants were men and 3 of them were women. |
| **Data collection** | | | |
| 17 | Interview guide | Were questions, prompts, guides provided by the authors? Was it pilot tested? | Yes, a semi-structured interview guide was used. It was pilot tested with two young persons that did not have experience with counseling. |
| 18 | Repeat interviews | Were repeat interviews carried out? If yes, how many? | Yes. 8 (one interview among each of the 8 lay counselors who participated). Two interviews were conducted using the same interview guide: one in November 2019 and another in November 2020. Only the repeated interviews (conducted in November 2020) were used in the qualitative analysis. |
| 19 | Audio/visual recording | Did the research use audio or visual recording to collect the data? | Audio recording |
| 20 | Field notes | Were field notes made during and/or after the interview or focus group? | No |
| 21 | Duration | What was the duration of the interviews or focus group? | Various lengths: 15 min to 60 min |
| 22 | Data saturation | Was data saturation discussed? | Not applicable |
| 23 | Transcripts returned | Were transcripts returned to participants for comment and/or correction? | No |
| **Domain 3: analysis and findings** | | | |
| **Data analysis** | | | |
| 24 | Number of data coders | How many data coders coded the data? | Two of the authors of this study (Charisse Ahmed and Amelia Van Pelt) |
| 25 | Description of the coding tree | Did authors provide a description of the coding tree? | Yes. The authors developed a codebook with a definition for each code. |
| 26 | Derivation of themes | Were themes identified in advance or derived from the data? | Derived from the data |
| 27 | Software | What software, if applicable, was used to manage the data? | NVivo |
| 28 | Participant checking | Did participants provide feedback on the findings? | Yes |
| **Reporting** | | | |
| 29 | Quotations presented | Were participant quotations presented to illustrate the themes / findings? Was each quotation identified? e.g. participant number | Yes, we used quotations to illustrate themes. Each participant was identified with a pseudonym to maintain confidentiality. |
| 30 | Data and findings consistent | Was there consistency between the data presented and the findings? | Yes (as represented by the quotes) |
| 31 | Clarity of major themes | Were major themes clearly presented in the findings? | Yes |
| 32 | Clarity of minor themes | Is there a description of diverse cases or discussion of minor themes? | Yes |
