# Supplementary material for: Implementation Determinants of Problem-Solving Therapy Delivered by Near-Peer Lay Counselors for Youth Living with HIV in Botswana: Lay Counsellor Perspectives
Source: Glob Implement Res Appl. Author manuscript; Available in PMC 2025 Dec 1. (PMC11905926; doi:10.1007/s43477-024-00126-6)
Supplement: Ahmed_Implementation Determinants of Problem-Solving Therapy S2 (Theme Development Matrix) [file NIHMS2051337-supplement-Ahmed_Implementation_Determinants_of_Problem-Solving_Therapy_S2__Theme_Development_Matrix_.docx]

**Theme Development Matrix**

| **CFIR Domain** | **Emergent Barriers and Facilitators *(Deductive and Inductive Constructs)*** | **Illustrative Quotes** |
| --- | --- | --- |
| Outer Setting | (-) Clients may not feel comfortable meeting for counseling in certain clinical settings. Clients may not favor the clinic location of the sessions as they fear that their counseling sessions will be shared with doctors *(Patient needs and resources)* | “They don’t really feel free in Baylor… because of the whole set-up…. But with [the peer counselors], when we are here they feel free.” (A17)  “The advice was that we asked that we hold our sessions outside Baylor so that clients could feel free. Because clients may feel like when we are with them in sessions we are going to tell the Doctors’, because here it’s just one family… They are going to feel that we are going to tell the Doctors’ how they live and what they say-what they say, though you have told them whatever we do-we talk about is confidential… The part that here they are not comfortable around them it’s that which causes clients to runaway most of the time without completing sessions, others not totally being honest on the whole thing when they are doing surveys so that they can pass and the person will not have to come here but then some-one client can tell you that “aaah, it will be more easier if we were somewhere else maybe at main mall or in a park”. You see?” (A18)  “almost every three months some they have spent most of their time here, always coming here, so they don’t really feel comfortable to be coming here attending sessions here you see?…yes we understand this was supposed to be a bench that where we stay we take a chair and have a seat but then the environment that we are here, besides we have another facility that side which is meant for the youth… the other side, across the road… there’s another facility for the youth with computer labs and everything like that but for them being here reminds them of how they live” (A18) |
| Outer Setting | (-) The intervention requires transport to the clinic, which is a barrier due to COVID-19 fears and/or restrictions – the need here may be education, offering virtual sessions, etc. *(Patient needs and resources)* | “I think it did in a way because uh less people are coming here, less people are...are not...its like more people are afraid to...to be moving around and they...they try to pri...to prioritise some things and they think this is not a priority to them. So they cant risk getting Covid 19 to coming here and get counselling. That’s all I think... I am only assuming that they are not coming here because of Covid 19.” (A11)  “Ever since Covid started...just this year in its self, I haven’t seen any clients… I haven’t seen any clients since this year started… it gets boring, it gets frustrating... because its now like...now that you not seeing clients its like whatever that you learnt...its like now you are slowly forgetting about it and now that you just come here for four hours not seeing a client or anything like that...” (A13)  “Counseling sessions currently, its-it’s not enough because we are not seeing clients currently, because of Covid-19 issues and yeah also the protocols aren’t allowing and also like the clients they are just scared... just as everyone is scared, imagine now contracting Corona when I had went for counseling… Imagine a parent hearing that I contracted corona when I went for counseling, in which its one of the challenges that when it comes to parents, some they don’t really like encourage their children to come see us.” (A18) |
| Outer Setting | (-) The intervention is not accessible for people with disabilities *(Patient needs and resources)* | “Most of them they are using transport…on that issue of transport… we have disabled people… we can’t really help them now… none of us have never attended those kind of disabled people… it’s our wish to attend [to] them because they also need therapy sessions but then one of the problem[s] that we discuss[ed] with our managers was that disabled people cannot come here because some of them need special transport to be here.” (A18) |
| Outer Setting | (-) The location of the counseling sessions are not ideal for people who live far or in rural areas. *(Patient needs and resources)* | “What I wish for it’s for us to go out there and….not only the city, even the rural areas they need counseling… the kids there they need counseling, they deserve it also… they don’t get a chance to see something like these maybe just one, day pop about it at any village and pitch our research and talk about these issues you know they will come out and talk to us” (A14)  “but they don’t really have a problem coming here besides the only hiccup they have is transport, because some come as far as Mochudi, Ramotswa, Tlokweng…” (A18)  “[Clients who live far] affects because some come- some can be late… some come late… They stay away in Molepolole… in Mochudi, so they use public transport mostly.” (A18) |
| Outer Setting | (-) The intervention conflicts with school hours | “…some are students they will come from-from school to come and coming in the afternoons.” (A18) |
| Outer Setting | Strategy: Implement virtually, in school, and in satellite locations (school implementation can also help with parental interference) | Debrief Sessions: “currently I would say umm with the corona virus issue I think some of us should be at home doing virtual-virtual meetings with them because always boarding combies or public transport, because most of us use public transport and it’s risky cause of the corona virus, though we are getting tested every two weeks but then it’s a must that as a person that ah at least we should change and attend at least one session virtually so that we cut moving back and forth and interacting with other people on the road.” (A18)  “[I wish] to go into schools… because we are only based in Baylor, go in the streets talk about friendship bench, people will know there is friendship bench I could go there for counseling.” (A14)  “So but one of the suggestions that we made was that for-if you have a clients as students, why can’t you go to school and attend them during lunch time or during their part time of the study time, so that then we avoid them coming here… and also going back because it’s-it’s it’s a responsibility along the way… and when the kid-when the-when the client comes you have to report to the parent that the child has arrived… when they leave the session, the child has finished the session you see? With us going to schools it will **cut the whole barrier** because a parent can call saying the child has not arrived on time…” (A18)  “going to schools will see-especially the children who are schooling, if we could go to schools and meet them in schools and come back, that is us they don’t have to miss school and stuff like that or missing schools it affects them also academically in their stuff so yeah.” (A18)  “…as the project expands umm we need to have bases or satellites across, for instance if-if Gaborone could know that in Baylor we are meeting all clients that in Gaborone not surrounding areas. For clients in Mochudi we have to have a satellite in Mochudi for in Molepolole, you know something like that so that we can accommodate people from Kweneng, from Kgatleng instead of all people from all different regions coming to one place you see? Maybe if we have satellites it could be much easier.” (A18)  “...I think it was better if we had satellites so we could meet them at least you know that if you-if you have a client in Mochudi I will be in Mochudi at 8 o’clock, I will be in Mochudi at 8 o clock and then meet my client wherever they feel free to meet. Because some they will-they can wake up in the morning from there to Mochudi on time, and you will hear them saying ‘but I woke up on time, but the only problem was that from my area to bus rank there was no taxi, so I had to walk 30minutes to bus rank’” (A18) |
| Outer Setting | (-) Frequency and duration of the sessions may not be enough for clients needing more therapy | “I think it depends on the situation… what we are talking about… some come with easy problems like ‘I am failing at school’… some say ‘I have family problems like not staying well at home… some are easy problems that you solve right away... the situations that cannot be-that are hard can be seen twice in a week like if you are saying umm they-they just knew about their HIV status and it’s not easy for them to accept themselves, I feel that’s when they can meet more counseling sessions ‘” (A14)  **“**It depends. The longer the session depends on the kind of er... situation you are facing with your client. The thing is there are clients that are very emotional. They will say be...Lets say during the...the...the course of the session, they will be crying. So [you] can’t tell somebody whose crying getting pain out to stop crying.” (A3) |
| Outer Setting | (+) Clients want more sessions | “[Clients want to meet more often] because at the end of the session, that’s when they realize the purpose of the-the study or the session.. because at that point they are now free you see? So they are always having that facial expression or smile hoping to come back.” (A12)  “But also them, they will tell you that for now I feel I am fine, after that session after third sessions-fourth sessions they will tell you I feel I am fine but some they will tell you “ I will like to meet you as time goes on” you see?” (A12) |
| Outer Setting | (-) Some clients want shorter sessions | “Umm up until the third session that’s when you will see, they would even tell you that ‘for now I feel I am good’” (A12)  “Yes but most of the time the clients when they get to like the third session, they say they are fine, but less numbers of them they would want to continue…” (A12) |
| Outer Setting | Strategy: Extend the duration of the sessions | “maybe 20 minutes could be added on top…” (A14)  “think one of the best things we should umm is to increase our sessions from six to twelve, from six sessions…” (A18)  “sometimes six sessions are not enough…sometimes six session are enough for some others so I think if we have-if we could have umm a written thing say our sessions should-should be from one to twelve and then it would depend on the client where they need to break and say here I think I’m okay I can now cut you into session four or session eight or session twelve” (A18) |
| Outer Setting | (-) Negative cultural views about counseling | “I don’t know about white but and-and Asian but then when it comes to a lot of black families there is this-this-this-this feeling towards psychology that’s there, you see? Where they feel like “Aaah I don’t want to go see a shrink” or “I am too good for psychology” or “psychology is only for people that-that is something wrong with them”, you see?” (A20)  “Experience that I’ve er....noticed from er... working with the youth basically is that er...most of the youth...er...we are currently going through a lot of... I would say life challenges that normally er...older people...older people yes! er...normally wouldn’t understand… because they...they would, they would think that just because you are a...a youth you don’t have a lot of things to be worried about.” (A3)  “Older people don’t believe that kids can be depressed, can have anxiety…” (A3)  “Even the likes of Substance Abuse. They don’t believe that kids usually do that and most of the kids that abuse substance they do it basically to...to try to overcome something that uh...that they don’t want to face head on...” (A3)  “So that’s the most challenging part. For them to talk to us, the thing is like, I don’t know how to put it. The thing is, when a kid... normally in our society when a kid, lets say someone is...when a kid says is going to see a Counselor, to our parents its like we are going to **reveal family secrets**. The thing is; That’s how it is… Lets say maybe the child is being bullied at home, is being er...abused and what, that process of them going to see a counselor even the parents will be on edge that this child may...may expose us yah!. So that kind of...of environment will cause that child to...to...to not say anything willingly. They just avoid most of the questions and answer them with short ended...short ended...ended questions to avoid er...revealing more information.” (A3)  “Me personally I just, I just keep on reminding youth...these youth that er... Since us we grew up in a...a society where we don’t usually shared our...our feeling, well our parents are not those kind of people were, they believe er...er...a child can be depressed, er...can have anxiety, can have this other problems because they think they are for older people only… They don’t believe the youth can have those problems.” (A3) |
| Outer Setting | (-) Parental disapproval of the intervention *(Knowledge and Beliefs, Other Personal Attributes)* | “: Challenges we had was getting them to open up. And also... having to ask them to come because we had this..uhm..client counsellor confidentiality, but then the challenge that we encountered most was that parents want to know what you were talking about. You see, that was a problem. So the big challenge was that after they told them, then it was like the parents told them don’t go there again.” (A17)  “Before they come for counseling… one needs parental consent... so the other parent will agree and the other one will not agree.” (A14)  So that’s the most challenging part. For them to talk to us, the thing is like, I don’t know how to put it. The thing is, when a kid... normally in our society when a kid, lets say someone is...when a kid says is going to see a Counselor, to our parents its like we are going to **reveal family secrets**. The thing is; That’s how it is… Lets say maybe the child is being bullied at home, is being er...abused and what, that process of them going to see a counselor even the parents will be on edge that this child may...may expose us yah!. So that kind of...of environment will cause that child to...to...to not say anything willingly. They just avoid most of the questions and answer them with short ended...short ended...ended questions to avoid er...revealing more information.” (A3)  “’Why my child needs counseling.’ Parents don’t intend to understand the importance of counseling… some parents are not open to us, they will say no she is fine while [when they are] going through a lot… Then [they] can’t tell [their] mum what [they are] going through so it’s best [if they] go tell somebody…” (A14)  “Counseling sessions currently, its-it’s not enough because we are not seeing clients currently, because of Covid-19 issues and yeah also the protocols aren’t allowing and also like the clients they are just scared... just as everyone is scared, imagine now contracting Corona when I had went for counseling… Imagine a parent hearing that I contracted corona when I went for counseling, in which its one of the challenges that when it comes to parents, some they don’t really like encourage their children to come see us.” (A18)  “Me personally I just, I just keep on reminding youth...these youth that er... Since us we grew up in a...a society where we don’t usually shared our...our feeling, well our parents are not those kind of people were, they believe er...er...a child can be depressed, er...can have anxiety, can have this other problems because they think they are for older people only… They don’t believe the youth can have those problems.” (A3) |
| Outer Setting | Implementation Strategy: Educate parents about the intervention | “Basically we...we try to talk to the parents also… We tried to show them er...er...what we really do… It’s not about getting to know their secrets. We just show them that all we want to know is to understand what the child is going through. Its all about him/ her… not what’s going on at home, what they are doing at home. No Its all about what she will...she/ he does during this kind of er...stressful er...time.” (A3) |
| Outer Setting | Some clients have competing needs that may get in the way of their participation in the intervention | “I have to be sensitive because l don’t know when was the last time this child had a meal, you get? So l didn’t have..l didn’t want to make the issue of finances to… Apparently for the youth…apparently these days its like money is one of the most depressing things for them… I had to work a way around that. Like for most of the clients that l had, that had challenges with the money, l had to come up with a way of how can l talk to them about coming. Because l remember the first time l saw a client and then she was worried about money, and then l had to tell her, remind her because obviously they sign a contract before we see them… I had to remind her that in the contract, you are going to be given P30 after l have seen you for that 1 hour. Then within this P30, you can discipline yourself that you… this money is strictly for coming here. If at all l touch it, at least let me have something to come here next week” (A17) – originally coded as characteristic of individual |
| Inner Setting | (-) Counselors do not feel compensated or valued enough for their work *(Learning Climate, Culture)* | “And it...it  has also uh! Uh....affected me negatively because I’ve...I’ve invested so much on...in this program but the...the rewards are...are not necessarily meeting my expectations…” (A11)  “I would like to be...to be compensated more for my time.” (A11)  “We talked to [our Project Manager] about it, that the combis have increased prices, and then she felt that she is already giving us way too much. Despite this thing being a volunteer programme, for some of us…normally volunteer programme you come and say l want to volunteer, but with this one, we were called…you see? We were called that there is a volunteer programme starting, we have put your name on the list. It’s starting on this day, be there, you see? So its not like we came like l want to volunteer, we were like okay, because they know we are home we are not doing anything, okay then…” (A17) |
| Inner Setting | (-) Counselors do not have enough money for transportation to the intervention site (which is related to transportation wasted on clients who do not show up for counseling appointments) | “I would like to say the...the capital. I know...I know its...its a voluntary work but uh in terms of transport, in terms of transport I feel like er....its...its...it is not enough and is... I think if...if...if...if they increased the...the...the...the rate we get a day, it would be much better because right now we are getting is it 70...? P70.00 a day...a day and we come twice in a week and the...the other day we don’ t get P70.00 but we get P50.00.” (A11)  “R: Right now we are getting P480. That was before the price increase of combis. Of which we did mention to our supervisor but she is… it didn’t change. You see?” (A17)  “ Imagine if I get a call like now to come here...this side, its gonna take me a long time, its gonna need money also. What if at that certain time I don’t have any money with me.” (A3)  “Basically money, money is a problem.. because er normally we bring...You know we have to get here... Like I said sometimes they will just pop up out from no where… I came to see my...my counselor and the counselor, like me I stay in Ramotswa... Imagine if I get a call like now to come here...this side, its gonna take me a long time, its gonna need money also. What if at that certain time I don’t have any money with me… You see that’s the kind of problem that we face.” (A3)  “The problem that we as counsellors we are complaining about is the money that we get… The money that we get is not even enough to support our own selves… Because as we’ve noticed transport went...transport fairs went up. The thing is a lot of things are expensive... Even for us just to wake up and come here when you know that...that’s...that’s a cost from your pocket. The money that you are going to get is not going to cover the amount of expenses that you are using to come and go back… That the other problem that we are facing… Most us because some of the counsellors end up not pitching.” (A3) |
| Inner Setting | Strategy: More compensation | “I should think because....because this is...this is...not a...this is not a job but a voluntary work, I should think P800 would do for...for transport and a bits of lunch money, I think that will be enough because er... we are volunteering its not like we...we are working.” (A11)  “R: Yah! Money! I know we never have enough money, it’s something that is universally known… but, just a little bit up wouldn’t hurt.” (A17)  “Compensated a bit because we have… some of us have children. We have children and depending on your parents to raise your children sometimes it’s not nice. You have to at least be able to pop out packed snack, lunch for the kids, a bit of juices for school, get them toiletry as well. You cant always be depending on your parent to do everything. So even if with the small P480 that l get l try by all means to…okay, l am getting my child drink and chips for the whole month, right? Budgetting like… the chips you get bulk, nik naks packs, then you have to buy toiletry, you half it, this time its roll on,next month its spray. You cant get all of it at once.” (A17) |
|  | Strategy: Increase clientele so counselors can make more money | “So if they were to increase that or...or increase the... the activities or...the...the...the clients in our program that...that could help... we only have one session a week and the other session is the family... family meeting where we have...where we discuss our problems and everything that happens during the week. I will suggest three sessions a week at least… If we could get more sessions, that will work.” (A11) |
| Inner Setting | (-) Some counselors do not trust their peers | “**…**we had a snitch amongst us. If I may say so...um just that we were like... if we have a snitch amongst us its gonna be impossible for us to like come in the group where we feel like its safe for us to open up and just fresh our mind... because that person will take whatever that we are talking about and then take is somewhere else. So that was a bit downgrade for it but then we ended up talking about it and then it stopped. So I would say that.” (A13)  “The only challenge uh I... the only challenge is that after the snitch appeared its more of like we withdrew from talking to each other... Its like we’ve nearly lost the family group that we have created, but um we somehow managed to pull through and just be together. But now its more of like whatever you say you have to be careful because now at the back of your mind you are like maybe even this person can snitch on me or something like that. So you always have to be careful of what you say now...” (A13)  “Yes, so some of us we can’t open because we feel when I say something somebody else from the group is going to say it out to some people and then tomorrow I hear it in the clinic” (A14)  “It goes out we are always-like last week we-we were telling her that our issue is still the same because we once talked about it last year, then it went better now it has started again like somebody is there being our spy. Whatever we say it, goes out.. So it’s a challenge, it’s really it’s too much it’s really too much, it’s like we don’t trust each other… as a family, we are a family now… People need to grow like people need to know that my personal life it’s my personal life, I might have spoken about it in the group because I see you as family” (A14) |
| Inner Setting | Strategy: Dealing with mistrust (discuss as a group and involve leadership for accountability) | “Uhhh... since there are now no rumours or whatever that the person used to do, I think its fine but if it happens that it comes out again then it will be best one on one of each person saying out their issues about that particular person and then we get into a group with our support lady and then [our Project Manager] and then we talk openly about it. I think that way then the person will see that this is really serious. So that will help.” (A13)  “We went to the support group and then told the lady that this is what is happening and then talked to her and she was like um if this continues, this is what’s gonna happen to you.” (A13) |
| Inner Setting | (-) Coworker conflict | “So some could up with uumm, for me personally I would say umm I had challenges whereby like umm me and one of the lay counselor we had to like get into a fight, because of certain-because these debriefing sessions I would say its free flow… if you have a grudge with someone, you just say "ah personally [this counselor] did not treat me well the other day”. And then it’s one of the issues where you will find that there are arguments but then arguments ensue looking at that the decision we took as a group the other day, it did not favor another individual. So those kind-so those are the only challenges, that there are arguments but it does not take too long before the issues are resolved.” (A18)  “Okay uuuh for-for one its-its-it’s not getting along with my other counselors or maybe counselor, let me not make them all seem as if they are bad, its only one particular person I don’t get along with.” (A20)  “She [has] been disciplined before, so she will be disciplined and then for that moment she will complain and cry and what not what not what not and then after a while she reverts back, so like I don’t particularly know how like the bosses could handle that or how another person but that can be like, that can be really draining.” (A20)  “And conflicts among us, yes! We do have conflicts like any other er group of youth. Just some disagreements, some debates there and then… On what we could have done, what we should have done. You see those kind of challenges, that’s basically it.” (A3) |
| Inner Setting | (+) Counselors feel supported by leadership (Leadership Engagement) – counselors made their boss aware of the issues they were having with money and transport | “[The Project Manager] is trying her best to try to... to try to uh...like try to solve that problem.... because uhhhh! Like last month...last mon...last month she suggested we translate somethings for a certain amount of money. Those...those activities, those kind of activities help because at the end of the month when you combine the all the money its a...its a bit better.” (A11) |
| Inner Setting | (+) Counselors have a positive relationship with the team (Networks and Communications) | “…I am grateful to our boss for, and I don’t think she can actually say I don’t respect her. I think I have told her many times that I respect her, I adore her and like-like I think she know that i-even though like we have this-this disagreement… I still hold her like as an amazing person, as like one of the best bosses a person could ever fee-could ever have…” (A20)  “"Interviewer: Yah! So, so what are your thoughts about the Lay Counsellors. Your team, your colleagues what are your thoughts about them? Interviewee: Ahh! Us we are perfect.”(A3) |
|  | (-) organizational constraints on changing the location of the session - Implementation Climate (but maybe even culture?):  (-) the counselors have limited decision-making power | “Yes [regarding the need to have counseling sessions outside of Baylor due to client preferences] which was supposed to be the initial idea but then changes because its partnerships-partnerships they have protocols that they agree on so it’s something that we keep emphasizing to the management to fix the issue that “let’s look for the best product of that the children want 1-2-3-4”... Let us not say that, “the other partners are saying this”, make them understand that the children feel free when they are outside Baylor.” (A18)  “[My boss] could be the one who at all you know, we could be seeing clients twice in a week regarding that issue… On the issue that they are having right there” (A14)  “ I would hope in the long run things will change.” (A11)  "Uumm for instance, for now like as we are here we are using facilities- Baylor’s clients, and Baylor’s facilities. So There are certain laws that governing us here in Baylor, so we have to like, that is if we are being oppressed we should understand that it is protocol, it was-it’s a must to be there. But you shouldn’t just wait and say you’re being oppressed, you should also talk to-you should know supervisors that at least If i could talk to this one they could help me better, because they understand me. Because if you don’t do that, if you don’t open up with some other things and they don’t understand you, they will never know if you are fine or not you see..” (A18) |
| Characteristics of individuals (Barrier #1) | (-) Diverse clientele have different personalities and needs which may hinder progress during sessions and can be difficult for the counselor to manage *(Other Personal Attributes)* | “The experience for me I think it’s overwhelming, because number one we have the chance to meet different youths from different backgrounds and different perspectives, the way they think, the way they see things and the way they behave so like I learnt a lot from them in different perspectives in terms of the behavior and their self-issues… And then also working different clients its-its-it’s some sort of like a problem also, because when kids they come-they come with different umm backgrounds… and then you as-as, me as a counselor I have to like adapt to different things-to different backgrounds” (A17)  “…even people, their personalities are different umm, I will give you an example; my first client ever-first client ever on the session on the umm bench...was a...aaah he was a quiet person. Totally-totally quiet like, their character if I can describe it, he was a quiet person, who was a native, a native child. That is like he was a quiet person and having issues of not opening up to me. That is there were no-you could see that this person has problems, they could talk much but because of the set-up of how they were raised as there are issues of family background… of how you are raised. They were raised as a quite child and that and that and that.” (A17)  “Umm the most difficult part is umm getting to know them, like to get to get them to relax and feel free because first sessions are always a bit tight. They don’t open up as much as you want, it depends on you as a counselor on how you approach them because yes we were taught on manual, on how to approach client and but we are encouraged to see like if you have a client you have to like notice if the client is talkative or is shy one, you have to find ways of dealing with them either they come as someone hyper or someone very shy you have to find a way around it so that you work together and then….” (A17)  “Like I’ve said, like during the...the session...the session the problem was the client was no...was...was, he was shy… He was not really the talking type. So, [I] had to maneuver my way around his wall to finally show him that ‘No! This is a safe space... you are free to talk... Whatever you wanna talk about.” Me I am just here to listen. So it took some while until I can finally bring down that wall that he...he build to protect himself.” (A3) |
| Characteristics of Individuals | (-) Clients usually do not open up during the are personal information, especially during the initial counseling sessions. Clients lack trust. *(Knowledge and Beliefs, Other Personal Attributes)* | “If a client doesn’t trust me, he holds back some information…” (A11)  “For me personally first sessions were always difficult across all the clients I had… even for others, for other lay counselors it is always difficult first like-first sessions, second session, third session, fourth session and fifth session is always smooth” (A18)  “they are af...they don’t easily open up to...to really talk about what they are going through.” (A3)  “The most difficult thing is getting somebody to really talk about their...their...their pain that they are going through... Like sharing their pain. What I would really...what really going on with...within their lives. That’s the most difficult part because during that process you are like opening up old wounds.” (A3)  “Umm I will mention one of them, repeated sessions are once, okay from experience, I once had a client who never really said anything. That is who wasn’t really opening up, across all repeated sessions and we then ended sessions just like that, we didn’t cover much though he had some…though he had a few list of things that should we-but then there were like 4 or 5 problems and then some of them we didn’t go over them” (A17)  “….they would see clients for the first session and then you will never hear from the clients again… they come with excuses and stuff… I: Oh okay maybe they-they were not warmed up to the counselor to open up. R: Yeah” (A12)  “The most difficult thing... the first session I should think is the most difficult because this person doesn’t know whether to trust you or not because of how fast the information can travel nowadays. So it’s a very difficult thing but as time goes, as the client [begins] to trust you it’s fine. The problem is when you start the session.” (A11)  “The advice was that we asked that we hold our sessions outside Baylor so that clients could feel free. Because clients may feel like when we are with them in sessions we are going to tell the Doctors’, because here it’s just one family… They are going to feel that we are going to tell the Doctors’ how they live and what they say-what they say, though you have told them whatever we do-we talk about is confidential… The part that here they are not comfortable around them it’s that which causes clients to runaway most of the time without completing sessions, others not totally being honest on the whole thing when they are doing surveys so that they can pass and the person will not have to come here but then some-one client can tell you that “aaah, it will be more easier if we were somewhere else maybe at main mall or in a park”. You see?” (A18) |
| Characteristics of individuals | (-) Clients may be reluctant to attend counseling sessions with counselors whom they have met out in the community *(Knowledge and Beliefs)* | “In most cases it’s not every youth or everyone that’s free to talk to someone their age… so most of the time… the clients that we see here know us… So in most cases when they find out that this person I always see them there in teen clubs and everything, they-they get reluctant in being free to be to us… Maybe it’s just the human mind… if I tell you something personal and I see you in public… automatically I will start thinking maybe you told someone or you are going to just say what I told you out and everyone hears it…” (A12)  “The first session that’s where I get problems where I have to make the client to feel comfortable when talking to you because some of these clients you get to meet, not necessarily meet but they get to see you so trying to talk to them when they had seen you before it’s a barrier.” (A11)  “I had one client I have seen him around because we live in the same neighborhood but me I didn’t know him. It’s easy for you to help someone you completely don’t know… At first he was not ...he was not comfortable with the...with the fact but after we had a conversation...after I asked him some few questions personal questions he...he...he got to relax.” (A11)  “Some of the clients, half of the clients I knew them… but I didn’t see them because I would say I ‘no I know this client, we have interacted from somewhere else, so I don’t want to see them… [give] my clients to somebody else’” (A14) |
| Characteristics of individuals | (+) Building trust and rapport with clients happens overtime as sessions progress *(Other Personal Attributes)* | “The easiest part is that umm...relating it to it here into Baylor, the clients here they tolerate more, what I really know-what I did found out is that they tolerate easier more with us cause I can have a client and then they open up to me and tomorrow I could see him with all of-with all of my colleagues but then we could see these people are now starting to feel free around us, it doesn’t necessarily mean when I’m their counselor they could just come to me and talk to me alone, they could also talk to other guys.” (A17)  “What I can say is challenging is to-to-to push our clients or me to push my clients to be open enough... because like we can spend about 10 minutes… going around the corner with the same question just to get the clients to be free to say something. So that’s the difficult part… But at the end it’s just them that just decide to just talk you know... sometimes after explaining everything they just relax…” (A12)  "So from that point I felt that because of the emotions she-she-she-she shows-she showed to me that means she is now becoming free and ready to say some things out so that’s where we picked up the session” (A12)  “I’ll say most of the clients that I saw.. there is a change in their behaviours because most of them they were literally shy. So ever since...like I keep seeing them out there and I can see that their confidence has gone from like 50 to like 100. So I am like wow! That’s really good because the first times most of them came to me they were...its more of they were afraid of talking or something like that.” (A13)  “They will see me these guys they really need to help me they are similar age why should I hide. And second sessions you would find that you meet a child saying that ‘aaah last time, first session I was really scared but now I’m very calm, I understand what you are talking about’” (A17)  “Seeing him being able to...to...to open up, to talk, to express his/her feelings, to...basically to...to give you some...the thing is basically they just need somebody to...to listen to...just talk to. So if somebody er...er...er... who started the first session they don’t really talk they expect you to talk to them, they don’t really then say anything. And then by our third session they are now the once who are taking control of the...the session. That shows that there is progress” (A3) |
| Characteristics of individuals | (-) Clients are not being truthful during counseling sessions *(Other Personal Attributes)* | “So our next session, it was kind of awkward with-like trying to find out what he really did. So he explained to me that “no honestly I did smoke so I-I didn’t mean to”...So it was challenging now because he-he I thought he told me what I wanted to hear…” (A12)  “You need to understand how they are because they are very tricky they can say they are fine but while they are not fine.” (A14)  “You could see that this child is saying that they are being abused back home but then in the actual sense you could see that this the person who is abusing other people… They fabricate problems, when they are the problem-that this person is the problem in this issue…” (A18) |
| Process | (-/+) Strategies for dealing with clients who are reticent or lack trust | “Interviewee: Okay, Ummm! There was one client that I had a difficult with, uhhh the first and the second sessions she was just quite. So I was like what’s the best way for me to like make her feel comfortable? Normally when we were in another room that side it was just too formal. So I decided to take just pillows and I asked her like “lets just sit down and pretend like you and I are friends”. So I made a conversation with her just...random thing but me asking random things I was actually using our... the paper...the questionnaires that we always use yah!. So I was actually using that and after I asked her all the questions since I wasn’t writing I was like you know what; thank you for opening up to you might...it might seem like you were not opening up to me but you actually said a lot of thing. Its like just the platform of the office just it not being colourful or anything like that you couldn’t open up so the fact that we sat down and talked like we were friends and she could open up. I was happy about it and then the third session that’s when I was like “last time this is what you told me” and she was like “oh! That’s a problem I didnt know” . So i was like yah that a problem so lets work on it and that how we went through it...through it.” (A13) … “She was happy about it because she was like there is nobody that she talks to. Its like she was the only child amongst them... and then she was staying with relatives and all that. So the fact that she was going through a lot all because of the relatives and all that she couldn’t open up to somebody. So her being able to open up to me, she felt good about it. And she always looked forward to coming to the sessions so yes!” (A13)  “Uhhhh! The difficult thing I would say is just one client that totally didn’t want to open up to me because she wasn’t really that free. So just me telling her lets just sit down and pretend like we are friends...” (A13)  “Like I’ve said, like during the...the session...the session the problem was the client was no...was...was, he was shy… He was not really the talking type. So, [I] had to maneuver my way around his wall to finally show him that ‘No! This is a safe space... you are free to talk... Whatever you wanna talk about.” Me I am just here to listen. So it took some while until I can finally bring down that wall that he...he build to protect himself.” (A3)  “The issue of them not opening up was for me… me to set up a platform where I can make them feel free that, this is just me and you… Well... tell them a bit about yourself…Or get them to tell you about themselves, what they are doing, school, what they like doing, hobbies, like open… get them to open up the mind, to make them open up., they should feel free… Yes, yeah that, we just had to do ice breakers.” (A17)  “Then-we just talk about it just that…maybe soccer or anything or music and stuff, then I go back…” (A12) |
| Characteristics of individuals (Barrier # 3) | (-) Counselors are emotionally triggered when they experience the same hardships as their clients *(Other Personal Attributes)* | “…You can have a client that is talking about [a] problem that I have experienced so that talking will open up some old wounds on my side and bring some flashbacks and all that” (A03)  “It doesn’t help anybody. So, the most important thing as I told you that you can have a client that is talking about er...problem that I have experienced so that talking will open up some old wounds on my side and bring some flashbacks and all that… So, that will cause...will stress me myself.” (A03)  “sometimes you can have-you may find where a client brings up a problem there that we face there but you have once faced it but then depending on your emotions on the time if they are too heavy for you….you have to find a way of not showing your client physically but say “can I please go and drink water… Or to even a point of changing umm the counselors, I could just tell my client I cannot deal with you for the reasons that I have which I will discuss with my supervisors and we are going to have someone new as a counselor but similar age, same thing whom will address you for the whole entire remaining sessions.” (A18)  “that is why I am saying we were taught all aspects that if things are like this you see, have to do one, two and three. And also the other issue in counseling, we have also our own separate counseling so that me too as a counselor I have someone that I am seeing that I am hoping to tell my problems regarding some certain issues that I am experiencing personal, either with my clients or my personal life.” (A18)  “Honestly um I’ve gone through what the client was going through so its more of like what she went through is like I put it...how do I put it into words...its like I had to pretend like I wasn’t going through what she is going through so for me to help them I had to like suppress my emotions.” (A13)  “R: YesI [I] am a person with emotions some they come with heavy issues and then I have to, i am a person I react but then they don’t have to see that I react I can make an excuse to go drink water but knowing personally that this issue hurts. Because some can bring personal problems that you once had in the past or that you are currently facing you see, that is why i am saying we were taught all aspects that if things are like this you see, have to do one, two and three. And also the other issue in counseling, we have also our own separate counseling so that me too as a counselor I have someone that I am seeing that I am hoping to tell my problems regarding some certain issues that I am experiencing personal, either with my clients or my personal life.” (A18)  “sometimes you can have-you may find where a client brings up a problem there that we face there but you have once faced it but then depending on your emotions on the time if they are too heavy for you… you have to find a way of not showing your client physically but say ‘can I please go and drink water’…Or to even a point of changing umm the counselors, I could just tell my client I cannot deal with you for the reasons that I have which I will discuss with my supervisors and we are going to have someone new as a counselor but similar age, same thing whom will address you for the whole entire remaining sessions.” (A18)  “you can meet a client… Who-who says something that will remind you of your own problems..” (A12)  “Uuuh for the triggering we are usually advised that, if you are brought with a case that is close to home, then refer to like either your colleagues or tell the supervisor or even tell the boss and they will refer to someone else you see? But for like I always told this in my initial training that...”that yeah… you have to understand that when you have cases that are close to home, when you have cases that hit close to home, sometimes it can happen that, you be the best person to help the client” you see?. So like that’s the only reason why I didn’t refer it, or like I don’t refer cases even when they feel like “eish I know a person that has went through this or like eish I have also dealt with this, or like my brother is going through this” so like I try my best to still be objective and still find -help the clients find solutions.” (A20) |
| Characteristics of individuals | (-) Clients do not always like to be referred to a different counselor if their initial counselor is emotional burdened by the topics discussed in the counseling sessions *(Other Personal Attributes)* | “…there are scenarios’ where my client will be talking about the problem that I am basically going through right now. But when I try to refer him to another counselor he doesn’t want that other counselor. He wants specifically to see me. You see where the problem is.” (A03) |
| Process | (-/+) Strategies for dealing with clients with same issues as counselor | “So I chose to put my emotions aside no matter how difficult it was. I chose that so that I could be able to finish my work and then after wards I took a break just... I did yoga before!... So I decided to do relaxing exercises and that help me to cool down and be able to see another client.” (A13) |
| Characteristics of individuals (Barrier # 4) | (-) Low self-efficacy among the counselors *(Self-efficacy)* | “…I work with very limited resources, I will say so limited resources were coming to say much from there, I have to use what I have so I could scale myself. But If I had more than twelve I-I would say things could be like different maybe scaling would be seven point five but I said seven point five looking at the fact that I attended twenty but out of twenty, fifteen were generals and then three were not attending properly and the other two broke during the sessions.” (A18)  “[I rate myself] a 7 because its above average... 7 is not a very high number because I feel like um I had haven’t reached that perfect level where I could say [I’m] fine with everything... if I could get more clients I think I would be perfect.” (A11)  “I think I will give myself an eight out of ten… because… most of the time I have seen h boys and then there were a time… I think maybe because the strategies that I would use with the boys I wouldn’t use them when I am talking to a girl… you will never know, so I believe like I still have to have ways or learn some ways that I can use especially on clients that are just quiet.” (A12)  “I will give myself 7.5 [out of 10 because] I am one of the unfortunate people that have never many clients, I have never crossed twelve clients and above.. so with the limited ones that I have most of them they were happy, or most of them about six of them we managed to conclude over six sessions…” (A18) |
| Characteristics of individuals (Facilitator # 1) | (+) High self-efficacy among the counselors *(Self-efficacy) – one counselor is good at compartmentalizing challenges* | “[I rate myself] 10 out of 10 [because]I learnt a lot and the fact that even up to now I still use whatever she taught me in my daily life activities is really good.” (A13)  “Rape [is a] topic that I haven’t went through... I don’t even know how to deal with it but just after talking to [my supervisor] I was like okay!... I can actually deal with this I can help her.” (A13)  “[I rate myself] nine out of ten [because] usually they come to me actually, they say we want to talk, so I feel [I am] just pulling them to me [without] calling them… they are very open to me and we can talk easily.” (A14)  “I think 11 [out of 10]… [yes] l am there… first of all, apart from being a person with depression as a counsellor l suffer from partial deafness so for me l find myself that l have managed to overcome, go all this way without limiting myself… So l feel that l overdid it, l did the best l could… others didn’t make the cut, then us we are here, we made the cut on how we handled the whole oral [evaluation]… We may not be qualified like Degree, Diploma kind of qualified, but certificate yes, we have experience of a year by now. Yah, we have an experience of a year… at some point in the future they should consider it for us it’s a job and then the others who will come, they can earn the 480’s. they can get the 480’s…” (A17)  “ I will rate myself 2000 [out of 10]… so the reason why I say that is because, look, I love all my co-workers and colleagues but I...the facts are just the facts, that I feel like it-it wasn’t on their part, it wasn’t for them to control but I feel like uuuh. Intellectual wise, like I work-my mind process works at faster rate than everybody else’s. Even work ethic, I don’t think that there is any person that can outwork me…” (A20)  “…as a lay counselor, with the information that we have been given and [with]the little window that we only inclined to work with, I would confidently give myself 10…. Because I feel like I constantly challenge myself , like I constantly watch interviews and like watch different ways of asking questions, different of ways of like-like seating, seating arrangements, different ways of making people laugh, different ways of like making a conversation better you see? Different ways of making a client feel better. And I feel like I do that the more than anyone else like I am constantly trying to make my sessions better with each and every client. So I’d confidently give myself 10.” (A20)  “: [I give myself an] 11 [out of 10]… Because from all the experiences that I’ve seen during **all this trying times..** [I’m] prepared that whatever any issue youth bring to me to talk about I’ll be...I am ready to talk, to help them out.” (A3)  “"Honestly um I felt like  it was... if may say crazy at first for us to be learning all this in such a short period of time but just after learning everything I was just like wow! Its possible its just that you have to tell your mind that this what I wanna do this is what I want to learn because as human beings we will always have to learn.” (A13) |
| Process | (-/+) Strategies to improve self-efficacy | “I think um more practice more clients...if I...if I could get more clients...I think I...I would be perfect… More practice more clients. I think We should get more clients...” (A11) |
| Characteristics of individuals | (+) Counselors believe that the clients find the intervention acceptable and appropriate because they have a chance to work with counselors around their age *(Knowledge and Beliefs, Other Personal Attributes)* (Facilitator # 2) | “There is no language barrier because we could communicate anyhow, because we are both youths so they could say whatever they need to say but then it is easy for me to understand. Either they use street language… I can also like get the message from that rather than someone who is older doing that, they won’t really get to know street language and other things that is happening on the youth.” (A18) – **originally coded as characteristic of intervention**  "Back then you would only be bullied in school physically nowadays you are bullied in school but on Facebook and instagram. So that is why I said working with them is much easier because we understand the same thing that they are going through, we are going through it, either I have went through it seeing it from that person or I experienced it personally, so it’s much easier to work on all issues around, even issues of sex and other issues, it’s easier to talk about them when I am with them because they open up….” (A18) – **initially coded as intervention characteristics**  “so it is easier for them when you talk about Facebook, we can talk about Facebook because I know Facebook, we can talk about instagram because I know Instagram…” (A18) – **initially coded as intervention characteristics**  “Not really the same, similar but then they are younger than me but then they can open up and see he’s not really that old but its twenties or early twenties but then they can open up” (A18)  “because now they open up they have their people. They will see me these guys they really need to help me they are similar age why should I hide. And second sessions you would find that you meet a child saying that ‘aaah last time, first session I was really scared but now I’m very calm, I understand what you are talking about’” (A18)  “It’s easier [for the clients to open up with the peer counselors than with others] because umm number one the age… I’m not really older than them… it’s just a matter of maybe ten years, so they can open up.” (A18)  “What worked is that…uhm..I didn’t be like old ladies, like old Tswana parents when you are very judgemental when you hear what they have to say. I just went to….even if l am old l went to their level where l am like, okay, you are 20, l am also 20, you see?” (A17) **– coded as intervention characteristics initially**  “….we take ourselves as one as the youth… So, as we much as we seen them and then as much as they see us, they see us as one and the same, people on the same level.” (A3)  “The thing is they don’t see us as their big brothers and we don’t look at them as their...as our younger brothers… We are all equal.” (A3)  “So, basically when, when we are us just us; youth there is nothing difficult to talk about. We literally can talk for hours about literally anything; relationships, er...er...er sex lives basically a lot of things we can talk… As long as its us youth they are...I don’t believe there is anything difficult to talk about.” (A3) |
| Characteristics of Individuals | (+) Clients enjoy their time with their clients and feel like they are truly impacting their clients’ lives; seems like clients want to come back (can also be related to intrinsic motivation for continuing with the Bench) *(Other Personal Attributes)*  *(Related to Facilitator # 3)* | " So just me being able to work within that short period of that time and getting a lot of information from her and also having her wanting to see me again, it was.. I would say a blessing! So yah… It was rewarding!” (A13)  “But most of my clients were just, they left here very happy. To the point in where by even after the whole sessions were don. Even when they see me in the streets they will be like “ oh! Wow! I wish I could see you again, I wish we could...I could have your contacts or something like that” but we are not allowed... I am not allowed to do that so I was like “ you could just keep see me here or whatever” because just them telling me that being around your presence and all that made me see a better version of myself and look... not just think of present but also for the future. That’s a confidence for me, for them I mean. So yah!” (A13)  "lay counseling is great, you have no ideas how many kids you are going to help, help their lives improve and like how many people you are going to like-like-like engage with and like how many people you are going to uplift because like, this one client wanted my number, I was really saddened by the fact that we were not supposed to give clients our numbers and like because like he felt like I was sort of like a big brother” (A20)  "what they should know is working with kids is not easy….but after learning-after learning knowing them its very fun and they will come up to you and open up so easily.” (A14)  “[Clients want to meet more often] because at the end of the session, that’s when they realize the purpose of the-the study or the session.. because at that point they are now free you see? So they are always having that facial expression or smile hoping to come back.” (A12)  “I enjoy working with the youth when it comes to counseling, I enjoy [it] very much.” (A20)  “...it was very exciting to work with young people such as me because it...it also shows me that all those challenges I go through its not only me. There is someone going through them and... and it’s also exciting because I get to help my peers.” (A11) |
| Characteristics of Individuals (or Intervention Characteristics) | (+) Counselors are personally invested in Friendship Bench and intrinsically motivated to stay in the intervention (they want to see it continue to grow) *(Knowledge and Beliefs and Other Personal Attributes, or Intervention Source and Evidence Strength and Quality, Relative Advantage) (Facilitator # 3) One counselor would avoid making referrals because he felt like he had to support his client (A20)* | “And it...it has also uh! Uh....affected me negatively because I’ve...I’ve invested so much on...in this program but the...the rewards are...are not necessarily meeting my expectations…” (A11)  “I think...I think time. Time...uh...I’ve invested a lot of time here and the time I could be... be using it to...to look for ways to be financially stable right now because right now I am not financially stable. Yes!” (A11)  " Yes! In a way but I’ll...I’ll...I will not say it has...it has derailed me from doing what I am doing here. Because er...like I said its a voluntary but yes I...I...I...its...its a personal choice to come here so...yes! I should think it not...it did not derail me that much but is...its a factor.” (A11)  “I would...I would still be here because I do believe in the program but uh! I would hope in the long run things will change.” (A11)  "If they come crying to me or anything like that and feel free to open up. I am like  'wow! that’s a bonus for me’” (A13)  “the best part was when another client of mine...like it was the first session actually and she just started crying and I am like “okay!” like I didn’t ask her so many questions she just told me everything from the word go and she cried and that I was like “wow!” like how can somebody just come and trust me like that and then they cry out of no where. It was...to me it was shocking but i was like okay maybe its...its I did something nice to her to the point in where by they trusted me enough to like open up and just cry and release the pain that was going on with them. So I felt good about that.” (A13)  “So just that after the whole sessions were finished and I saw a different version of them I was like that’s something that’s something I can live with, it makes me happy that I can be able to help them to that extent.” (A13)  “[The best part of working with the youth is] being-being able to teach them how to solve their own problems, being able to teach them how to solve their own problems, being there for them, elevating them, uuuh-uuuh just just strengthening their confidence level uuhh and meeting new energies and and just-just the mere fact of being able to like being able to listen to someone, its-its also something I-I like seeing from their side like yoh they don’t have anyone to listen to them like their grateful that I took the time to like listen to them.” (A20)  “So like I thank-I thank safe haven and our boss as well, that is something I don’t think I could have-that is a skill I don’t think I could have been able to develop on my own and I thank this place for it. And I think that in black families and all black families that’s important.” (A20)  "The best part is like we have uuhh. We have three to four sessions with the same client and the best part is uuuh every last session we-we can see the difference between how you communicate with the client...and how they-their facial expression… you see that they are now free and seeing as someone they can do it... so that’s-that’s the good part of… like you are really doing something to help someone... [its rewarding]” (A12)  "Interviewee: So I called them to the corner and I was like “talk to me what happening?” and they were like shocked. They were like “what is going on, what are you talking about?” I was like for somebody who is going through depression um depression and stress this is what happens. You are showing those signs, so talk to me what’s happening. And they talked to me they opened up. They didnt tell me a lot but they told me the major points of what is happening. So just them being able to like trust me and telling me what was happening and I could help them see...find a solutions to dealing with their problems, I felt like that’s really good for me that...its good because even if I am not here I can actually help somebody out there. And after the...they...they found and solution and everything like that they came back to me and they were like how did you know that those were the signs of this? I was like this is what I am doing at Marina and they were like oh! Okay! That’s nice then you like but with how you advised me and helped me at least now I saw that I was about to make a bad decision of which it was going to harm the ones that are closer to me. So thank you for that! And I was like you are welcome! So that made me feel like just being part of Lay Counselling I can actually help somebody even if I am not actually here. So I really appreciate that. I really appreciate that the fact that they made sure that I was part of the group so yah!” (A13)  " I’ll say most of the clients that I saw.. there is a change in their behaviours because most of them they were literally shy. So ever since...like I keep seeing them out there and I can see that their confidence has gone from like 50 to like 100. So I am like wow! That’s really good because the first times most of them came to me they were...its more of they were afraid of talking or something like that.” (A13) |
| Characteristics of Individuals | (+) Counselors believe clients need the intervention (Knowledge and Beliefs) (related to Facilitator # 3) | "…its kind of stressful to...to...to be seeing somebody...somebody er...same age as you going through a lot, a lot of things that he/she doesn’t know what to do...But just that we are there, we are available for them it...it shows them that...well! it shows even us that these kids they need this kind of help. They just need somebody to talk to, somebody who wont judge, who wouldn’t get mad at them, who will be patient…” (A3)  “Yes! I still have that drive because I still... I still believe that there are a lot of youth out there that are confused...that are...that need help; our help.” (A11)  “I would like to see the project improving. The... the project being able to...helping a lot of youth out there..I would like to see that. I would like to...to...to see this program being all over Botswana.” (A11) |
| Characteristic of Individuals | (+) Counselors are growing personally from participating in the intervention (Knowledge and Beliefs, Other Personal Attributes) (Facilitator # 3) | "Interviewee: so what I learnt...the...the relaxing un exercises that we did, things to say to clients when they are crying or something like that. I used them on myself as in...not as a lay counselor just as me... That has helped me to deal with my emotions and be at peace with everything.” (A13)  “Now I can open up to my family I am still on that process but… it’s a confidence booster for me.” (A13)  “Ahhh.. I would like to say it has made me a better person because some of the...the things I used to do to...just to...to try to supress my stress... this program has shown me that they do not help but they...they prolong the stress.” (A11)  “R: Better person actually. And it is easy for...for…for..like I have a friend who at one point tried to commit suicide, so l could tell red flags because we were also taught about red flags to a point where l already knew she already did it, and l was like you know what, l am calling the police, they will get mad at me at some point, but l am calling the police. They managed to attend to her and took her to the hospital and drained her out. And they were like, if it wasn’t for me knowing what to do at that particular point, but she is in Tlokweng, l was in Partial,” (A17)  “...Even myself as a person had my own, so whatever I managed to gather around our whole sessions either ours as lay counselors or client’s sessions and other stakeholders, it builds me. It raised me to become more responsible to take time when making decisions… to observe what is going on when I’m making a decision, what error could I possibly encounter in future like monitoring is very easy a decision me too, even to open up and to ask for help even from a stranger, I can do that because I have that confidence.” (A18)  “Oh yeah, so like I...I-I cater that-that that this thing like...like-like its changed me in an enormous way that Uncles and some Aunties and like friends and like-like-like yeah, everyone like comes to me and like even like people like, we may work with in the clinic but not necessarily work with in safe haven. Yeah so like those people come to me and talk to me and then they like offload their issues unto to me and like they know that what they tell me is secured and It will never be leaked out” (A20)  “I think I am one person who gets angry- I am one person who gets angry and one thing I have learnt is to calm down, to cool and think before I act.” (A14)  “I would like to say it has made me a better person because some of the...the things I used to do to...just to...to try to supress my stress... this program has shown me that they do not help but they...they prolong the stress. And it...it has also uh! Uh....affected me negatively because I’ve...I’ve invested so much on...in this program but the...the rewards are...are not necessarily meeting my expectations.” (A11) |
| Process | (-/+) Publicize the intervention (Implementation Strategy) | “What I wish for it’s for us to go out there and….not only the city, even the rural areas they need counseling… the kids there they need counseling, they deserve it also… they don’t get a chance to see something like these maybe just one, day pop about it at any village and pitch our research and talk about these issues you know they will come out and talk to us. That-that’s one thing I wish for. And even here, to go into schools…because we are only based in Baylor, go in the streets talk about friendship bench, people will know there is friendship bench I could go there for counseling… to reach more people… I want it to grow and grow and grow and kids will also get in the groove and teach others or you know be counselors also… suggestion also that’s to teach those leaving school, form fives’… : so that we are not always going to be here we also need to know we have to step out and they step in” (A14) |
| Intervention Characteristics | (-/+) Going deep on issues can be taxing and requires patience | “Getting into that details sometimes can make the child lose the whole thing along the way, feeling like you are digging too much into their background and you will hurt them you understand. But then it’s one of the procedures that you have to do it you have to explain to them that you are going to keep asking them questions, they will be tiring but be patient with me cause we want the solution so that when you make a decision,, you make a very good decision understanding the whole background of your problem” (A17) |
| Intervention Characteristics | (+) Counselors believe the intervention is a good idea (Intervention structure) | "I think Friendship Bench is a very good idea.” (A3) |
| Intervention Characteristics | (+) the once per week meeting times work for clients | “Basically it works because we give them time to go test that...their...their...their...their....their...their progress with uh., the thing is, since they are going through a certain kind of problem… and then they will be given us solution... from those solutions we help them pick the best solution that they think it will work for them…. So during that process of...of...of booking them for the next week Thursday during that time period they are given that...that time to go work and try solutions. To go and work on that solution that he choose...” (A3) |
| Intervention Characteristics | (+) Counselors believe that the intervention is helping adolescents, leading to positive changes (Evidence strength and quality) (Facilitator # 4) | “So just that after the whole sessions were finished and I saw a different version of them I was like that’s something that’s something I can live with, it makes me happy that I can be able to help them to that extent.” (A13)  “them telling me that being around your presence and all that made me see a better version of myself and look... not just think of present but also for the future. That’s a confidence for me, for them I mean. So yah!” (A13)”  “they found and solution and everything like that they came back to me and they were like how did you know that those were the signs of this” (A13)  " because just them telling me that being around your presence and all that made me see a better version of myself and look... not just think of present but  also for the future. That’s a confidence for me, for them  I mean. So yah!” (A13)  “"The best part is like we have uuhh. We have three to four sessions with the same client and the best part is uuuh every last session we-we can see the difference between how you communicate with the client...and how they-their facial expression… you see that they are now free and seeing as someone they can do it... so that’s-that’s the good part of… like you are really doing something to help someone... [its rewarding]” (A12) |
| Intervention Characteristics | (+) The counselors view the intervention as better than receiving counselors from older adults (Relative advantage) (Facilitator # 4) | “R: For…I think for me, yes but then also the clients, because in sense of that umm some don’t really know our faces and it’s a new thing for them and then, overwhelming not in a bad way, in-in an odd way in which It encourages people like to talk more and then stuff like that, because it’s-its first time to have seeing some sort of this setup uumm because in junior schools where that-where they could get help its older people, older people I mean like parents but then they are counselors but then they are older and then for us to help us this fun of activity is-is one of the good things for them as they can now open up in terms of the language barrier, it is easy to communicate and then also age is not feeling much difference and those kind of things.  I: So you are saying it’s a good experience because the language barrier, there is no-is there language barrier or there is no language barrier?  R: there is no language barrier because we could communicate anyhow, because we are both youths so they could say whatever they need to say but then it is easy for me to understand. Either they use street language, I can also-I can also like get the message from that rather than someone who is older doing that, they won’t really get to know street language and other things that is happening on the youth.”(A18)  “…at my age when I was at junior school… I knew that the only way I could get counseling-professional help… is if I go to umm the society counseling people or I go to school… which is totally different for me and its totally uncomfortable for me… because i will be going there with a relationship issue and is not-I am not going to open up to someone older than me… because those are also almost closer to my mum’s age and then I can’t do that because basically I can’t do that at home with my parents or any of my guardians, so with that coming-for them coming to us is more easier… It’s easier [compared to the previous counselors they would go to]… at school and [community]…and also being here in Baylor…we also have umm senior setup” (A18) |
| Outer Setting | (+) The intervention aligns with cultural views related to age (related to Facilitator 2) | “rather than talking to an elder, you know in our culture era we can’t talk to an elder about sex issues, sex and staff like that.” (A18) |
| Intervention Characteristics | (-) Some counselors believed that the training was inadequate (Complexity) (Barrier # 5) | “I felt like the training though it was enough, it was not really enough… Because there are things that [we] face that we didn’t really train for.” (A03) |
| Intervention Characteristics | (-) Some counselors thought the training covered too much information in a short period of time, thus raising concerns about their ability to retain and apply what they learned (Complexity) | “Honestly um I felt like it was... if may say crazy at first for us to be learning all this in such a short period of time but just after learning everything I was just like wow! Its possible its just that you have to tell your mind that this what I wanna do this is what I want to learn because as human beings we will always have to learn. Like is just... I think it was… The short period of time that we had to learn everything about Lay Counselling.” (A13)  “This is my personal view, I always seem like it should be longer or at least the time-the time frame of 7 days is like a short time for that length of-of-of-of information” (A20) |
| Intervention Characteristics | (+) Some counselors felt the training was beneficial (Evidence Strength and Quality) | “[The training] was very worth it because some of the things I thought they were...they were okay and they are not okay…. for example… suggesting a solution to a client [is] not okay… You help a client to suggest solutions for himself… So overall… the training was very effective.” (A11)  “It was easy from... to...to switch from theory to practical because...or whatever...whatever we did during the...the...the...the...the training that’s what we came across when we are dealing with clients.” (A11) |
| Process | (-) Counselors believe that they are not getting enough clients | “It has now the problem being the clients...the liability of the clients. The clients are not showing up or there are no clients at all.” (A11)  “It has been a concern since...I...since...because at first we had...we had clients coming in. Then the number of clients started declining as we progressed.” (A11)  “I think what we really need is clients, we are not having clients, we are only seeing clients from Baylor we can’t see clients that are from outside.”(A14)  “Since-she needs to write a letter to ask if we could see clients from outside... which is making us struggle now... It’s very hard to work now or to talk about issues of clients… And not here only, outside… because people are-people want counseling… and we can’t refer them to friendship bench because they are only seeing clients from Baylor” (A14)  “And then as time went on, when we didn’t see any clients because it took a while for us to gain any-any momentum.” (A20) |
| Process | (-) Clients have issues with attendance or coming when not expected or show up late (maybe this is more of an outcome) which is tied to money | “ l have seen like 4 patients, 4 to 5 of which of those 5, which 2 stopped coming… The reason being that for them you never know what…why most of them stopped. But then l heard that there was this excuse that they didn’t have money, which was actually very funny for us because every time after we see them we gave them transport money…” (A17)  “It has now the problem being the clients...the liability of the clients. The clients are not showing up or there are no clients at all.” (A11)  “The challenge is always like coming here and also rescheduling issues because its-it happens, because there are issues of whether other commitments either from me or my client… of course because maybe I will come here knowing my client will be here at 2 o’clock and then at half past 2 they will be telling me no I have family issues… and then we reschedule to a different date which accommodate my client with me now, and I have to make sacrifices for my client because I understand it’s one of the things that we were told about. That sometimes you are going to have difficulties that you are going to invade some of the personal, private time.” (A18)  “Then you have to come. Sometimes you appoint with they don’t show up… They don’t show up.” (A3)  “…sometimes [the clients] will just pop up out from no where. I came to see my...my counselor and the counselor, like me I stay in Ramotswa... Imagine if I get a call like now to come here...this side, its gonna take me a long time, its gonna need money also. What if at that certain time I don’t have any money with me.” (A3)  “[The schedule] never benefited me at all… Basically they...because we work, they...they come at their own time… They can just come any day and be like “I want to see this guy” and I will be forced to come from home to come see him.” (A3)  “It wasn’t working . It doesn’t work for us. Even when you book a specific date, that okay! Lets say we meet tomorrow on Friday around 3. she will be like “Okay! No problem” then tomorrow they doesn’t pitch. They will pitch lets say on Monday they will be like “ I am here looking for you” and you will be like me I am not there then you are forced to come.” (A3)  “.because uh... I was talking about time, there is a problem of time management between us and uh the...the clients. Because at first when we started the program we used to like...we used to be called on hold...well, call on hold if you understand. Uh! Where a client will come then you will be called to come at a certain time so that you can meet up with this client. For example; if...if a client...if we decided that we should meet with the client around one, then the client decides to come around two... Its a problem because I do have other commitments outside there. So waiting an hour for a client...is...its a problem.” (A11)  “Recently, okay! Let me see say this, since March we haven’t had clients. Okay! I haven’t had a single client but before the scheduling was okay after they...they introduced the Blocks but before...before the Blocks it was...it was a problem because sometimes you could agree with a client to meet around 2pm and you, you would know that 2pm you have to be here but the client is not here around 2.” (A11) |
| Process | (-) Recruitment of clients is not happening fast enough (people may not know about the intervention and those responsible for screening clients may be overwhelmed with other responsibilities) | **“**there has been slow in...in scanning for clients or...or most...most of the people...most of the youth out there don’t know about...don’t know about our program… I do not know their names right now but people who are responsible for...for...for scanning clients... screening.... for screening clients… I think probably its not...its not the only thing they are doing here in Baylor. They have other obligations so I think that’s probably why.” (A11)  “then we mentioned why we can’t recruit people (Clears throat), they need a qualified person to do [recruitment]…like there is only one person who recruits clients, because we were talking about not getting clients at all which is not good” (A14) |
| Process | Strategy to address low clientele: provide more education in the community and allow peer counselors do the screening | “…increase where we...where we look for clients or we should scout more for clients at other places... if we could go around schools, if we could go around communities looking for...for...for...for people who show signs of depression or anxiety, that...that could help...” (A11)  “Yes! Well, Okay. I wish uh they could indulge us more uh because uh counselling clients it is not enough and we should...I think we could do more… Um, first of all we could...we could advertise the...our program on Facebook, we could have adverti...advertisements with our pictures in it. We could have videos we could have...we could go out around the community trying to...to scout more trying to help educate parents not necessarily children or the youth. We could...we could educate parents everyone around the community on mental health.” (A11)  “they have to engage us more. We...we could...we could do the screening, we could do the advertising... As the Lay Counsellors.” (A11)  “I think what we really need is clients, we are not having clients, we are only seeing clients from Baylor we can’t see clients that are from outside.” (A14)  “because we are only based in Baylor, go in the streets talk about friendship bench, people will know there is friendship bench I could go there for counseling.” (A14) |
| Process | (-) Clients are not being transparent about their availability | “In terms of their schedule? Umm maybe if umm I think for them is if they open up and say “On Wednesday I can’t meet you” and then they say “I can only meet you on Thursdays”. It’s one of the things that we have to ask the client that when exactly do you think you are free. So that we could meet, so if the client is free on Wednesday and then Wednesday I am held up, then that’s when I could tell them that “ Wednesday I am held up but Thursdays I am free from this time and this time why can’t we agree that we are going to attend our sessions in Thursday or if you have anything else you could just call us and then say “ I can’t make it on this day”, but they don’t really have a problem coming here besides the only hiccup they have is transport, because some come as far as Mochudi, Ramotswa, Tlokweng, some are students they will come from-from school to come and coming in the afternoons.” (A18) |
| Process | (-) Client schedule conflicts with school | “Most of the youth that we were seeing were students… So at times it will be difficult for them to come because they will attending lessons or something like that. Or even if they come they will come an hour later or something like that so that means there will be a pressure of time so that they can actually go back...go back to school or either go back to their homes . So I... I wish there was a way that we could like make sure that their time and ours could work for both us so they don’t feel pressurised and all that” (A13)  “It affects them in the sense that they are students, most of our clients are students, I will say so, umm it affects them because some they have to come in the morning…for our sessions...” (A18) |
| Process | (-) Counselors have schedule conflicts | “It was tricky because since am working and then they would want to see me at 4 while I knock off at 4… Yes so it was….the time were clashing… so I would try and make sure I see them at least at lunch time.” (A14)  “it was one of the most troubling things because umm a tertiary student… yeah because I have to like fix both of my schedules looking at what I have at school because it’s my first priority... So yeah but then working with them here it wasn’t really, the only hiccup was making schedules because I would take about a week or two to make my schedule here for counseling because umm I will be waiting for the timetable which always comes late at school…so yeah that was the only challenge, but then besides after the whole timetable issue is okay besides there’s nothing much I can say. So that’s all.” (A18) |
| Process | Strategy to help with client scheduling (the block system) and Saturday sessions and sessions in school and full-time workers | “they have come...come up with a system of Blocks. Where you are given four hours period to wait for a client, then the next...the next four hours they bring another counsellor.. since March we haven’t had clients. Okay! I haven’t had a single client but before the scheduling was okay after they...they introduced the Blocks but before...before the Blocks it was...it was a problem because sometimes you could agree with a client to meet around 2pm and you, you would know that 2pm you have to be here but the client is not here around 2.” (A11)  “There is somebody else who is already notifying the clients to come so if its my day to be on the block... I will be seeing those clients. And then if my block hours is finished and then somebody else takes over. Then they will see the clients when they are available.” (A13)  “I think working-seeing clients on Saturday could work also, like since am not working in Saturday… Saturday in the morning or lunch time… I think it’s flexible also because Saturdays usually have morning studies at school… they finish at 10…. then from 10 till 5 they are free…so that’s when they could be-use that time to come this side for this-for their sessions… from 8 till 3 [on Saturdays]… Transport is available during Saturdays. Sundays not all of-all the combies are working, just-I would say half but Saturday it’s a must, combies should be around.” (A14)  “for now to me its fine because we work with blocks. Everybody has their own block like l was on a block this morning, from 8 to 12, so with that…for me its fine because I know if it’s this day and l am not on the block, then there is somebody who is on that block. If its Tuesday mornings, Tuesday 8 to 12, and Thursday 8 to 12, that is when l know that l am on the block” (A17)  “Yes but then we resorted to having, to come here-for me to come here for the first two hours and then go to school, then later on I come for the last two hours because what we are doing currently, we are, we come here once a week for blocks… for our blocks or our counseling sessions… so they take four hours. So umm four hours which is straight up so which I did-I did divide that four hours into two, I come first for the two hours and then later on after school I also come” (A18)  “one of the suggestions that we made was that for-if you have a clients as students, why can’t you go to school and attend them.” (A18)  “Basically since what we are doing is not like fulltime… Cause that’s...I think that’s the major er...er gap that is causing us er... er... this kind of problems… The schedule is not working because if we were...we were...were here full time there wouldn’t be such er...er...er...things like that. Because er when the client pitches obviously he is going to find you here there is no where to be… So it will be easier even for those who, who come at a different date from what you appointed... It will be easier for you to attend them. Rather than for them to be waiting here for you while you get a call that you have a client, a client is waiting for you and then you take 30 minutes traffic... just to get here... And attend a client.” (A3)  “I think the solution that we...we need is basically to be...to have a... lets say a base. Somewhere...just our own space. Where we will be there full time to assist whoever comes whether we know him, whether we don’t know her. Just what ever help they need at a specific time they...they will know where to find us.” (A3)  “ if you come for the block from 12 till 4 that means you will be available throughout the afternoon… same as for the person who comes in the morning, they will be here throughout the morning” (A12) |
| Process | (+) Clients feel supported during debriefing sessions | “Uhhh! They were very helpful. Um more especially after I went through a case a of the girl who was nearly raped. So that was helpful for me because I was like...people are going through a lot they come to us for advice so if they come to us for advice I also need to get counselling because I was like what was happening to her its like now it was me. So its like I needed somebody to open up to so they can show me that this is what we need to do on everything like that. So just having somebody to open up to...” (A13) – **vicarious trauma**  “We could talk about different issues that we face with our clients and then we talk and they could say “but my client told me that when they are stressed they play football, what does yours do?” Those kind of things and then we discuss solutions around, that maybe if they are giving you a hard time why not do this and that and that and that. So it helps us as well to motivate us to wake up and stuff like that” (A18)  “I would say I am satisfied with the sessions a lot because we...we all feel comfortable talking to...to Dr Brooks. Some of the concerns we feels comfortable talking to them. Uh! Yes! I should think...I should think they are fine.” (A11)  “Yes it’s enough and really helpful because uuhh it doesn’t really has to be about the client because you can meet a client… Who-who says something that will remind you of your own problems.. So even us when the UB counselor comes, we just upload.. offload uuhh everything to her so it’s really helpful. It’s not always about the client” (A12) |
| Process | Strategies for dealing with vicarious trauma | “Uhh! In the groups I learned. There were some breathing exercises that we did, there are some other activities like the lady kept us engaged so we could always learn how to control our emotions and keep cool when we experience such difficult cases. So that to me it was helpful.” (A13) |
| Intervention Characteristics | Counseling sessions for peer counselors should be longer | “I would say the only thing is that we are seeing her for a short period of time… Its like we need more time with her but because she has somewhere else to go, her schedule is tight so that’s... somehow impossible but just having her around it really helps us a lot.” (A13)  “We see Pertunia, so normally with Pertunia’s sessions, we feel 2 hours is too small because now that’s her counselling us as a group. So now it’s like everybody has a story to tell about their lives and then she has to respond to them and she also has to teach us. So we always feel that the 2 hours is too small. We didn’t get the chance to say something some of us.” (A17) |
| Intervention Characteristics | Longer counseling sessions | “To about 3…4 hours depending on… With the meal there and there it’s because when we leave the houses, right? We know we are going for 2 hours, we need to know we are budgeting our stomachs for 2 hours, right? When if we are gonna make it about 3…over 2 hours then we should at least have a snack.” (A17) - counseling with Pertunia, |
| Process | Strategy: Anticipatory guidance | “The one for sex, we had to.. I mean it’s youth, they are growing up so you can’t tell them, “don’t do it”, so for ladies, girls, l had to suggest that, even though l knew the parents were going to get mad at me, but l had to tell them that there are contraceptives. If you feel that you cannot stop yourself from doing it, fine, like the clinic itself it offers contraceptives, we have depo, we have the pill, we have uhm… condoms…yah.” (A17)  “R: for drugs l had to…not to…I wouldn’t say be judgmental or give advice, but l had to put it in the doctor’s perspective for them to know that, as young as they are these small lungs are still developing, you get?” (A17) |
| Process | Strategy: Learning from others | “I wish that we could have like um other conferences sort of saying... and So that we can learn from other people because its just us...so just us having other  input from different organisations and something like that I think it can be of good benefit to us.” (A13) |

**Reflective Notes**

**I. Characteristics of Individuals**

**Barrier # 1:** Clients are reluctant to confide in the peer counselors, especially during initial sessions. However, trust and rapport develop as the sessions progress.

- **CFIR Domain:** Characteristics of Individuals
- **CFIR Constructs:** Knowledge and Beliefs about the Intervention, Other Personal Attributes
- **Emergent Codes:** Barriers, Reticence and Lack of Trust, Client Preferences and Attitudes, Dishonesty
- **Summary:** Reasons for lack of trust or reticence during the sessions can be due to personality traits (clients who are naturally quiet and shy), and clients who have seen the counselor in the community and fear that the counselors will disclose their personal information. Some clients were dishonest during sessions which may also imply lack of trust or poor confidence in the counselors. Although building trust is usually difficult during the first sessions, trust and rapport usually improves as the counseling sessions progress. Therefore, rapport building is needed to break this barrier. Parent perceptions can influence participation in the intervention. Two counselors suggested that clients are reluctant to attend counseling sessions with counselors whom they already know from the community. If clients they are counseled by someone they already know, their personal information may be disclosed outside of the sessions. However, in most cases, these concerns, especially getting clients to open up, resolve overtime once rapport and trust is established as the sessions progress.
- **Emergent Implementation Strategies:** Alter communication style to make clients more comfortable to engage (e.g., “pretending like we are friends”, icebreakers, reassurance that this is a safe space)

**Barrier # 2:** Parents may not approve of their child participating in the intervention.

- **CFIR Domain:** Characteristics of Individuals (or Outer Setting due to cultural norms around counseling)
- **CFIR Constructs:** Knowledge and Beliefs about the Intervention, Other Personal Attributes
- **Emergent Codes:** Barriers, Family disapproval
- **Summary:** Clients may have a discomfort or fear of disclosing personal information during counseling sessions due to family hesitancy around counseling such as fear of revealing “family secrets” (fear of disclosing is related to Barrier # 1). Parents may also not allow clients to come back for counseling. Another issue that may arise is that one parent may approve of their child participating in the sessions while another parent may not.
- **Emergent Implementation Strategies:** Educating parents about the importance of counseling for youth.

**Barrier # 3:** Some counselors experience the same hardship as their clients, which makes it difficult for them to continue with the counseling sessions.

- **CFIR Domain:** Characteristics of Individuals
- **CFIR Constructs:** Self-Efficacy, Other Personal Attributes
- **Emergent Codes:** Barriers, Emotional Burden, Navigating hard topics, Client preferences and attitudes, Confidence or self-efficacy
- **Summary:** Many lay counselors have experienced the same hardships as their clients and are unable to continue sessions if they become emotionally triggered by a shared experience with their client. This is related to self-efficacy since difficult topics affect their level of confidence in delivering PST. Some counselors still took on the emotional burden of counseling as they felt it was a moral obligation to support their client or because the client did not want to be referred to a different counselor. Clients deal with same hardships as their clients, but then have issues making referrals and may feel obligated to continue providing counseling (which speaks to how invested they are)
- **Implementation Strategies:** Stepping out of the room to get a class of water, concealing their emotions during the sessions, and later addressing them during an individual or group counseling session

**Barrier # 4/Facilitator # 1:** Counselors have varying degrees of confidence regarding their qualifications and level of competence as counselors.

- **CFIR Domain:** Characteristics of Individuals
- **CFIR Constructs:** Self-Efficacy
- **Emergent Codes:** Barriers, Navigating hard topics, Confidence or self-efficacy
- **Summary**: Counselors may have a low or high level of confidence in their ability to provide problem solving therapy to their clients. However, there may be difficult topics or situations that arise which counselors may not be prepared to handle such as rape, suicidal ideation, family issues, or issues around HIV disclosure. On the other hand, some feel confident in managing their role as counselors as they feel prepared and able to perform their role as counselors due to adequate training and experience while some do not feel as confident. Some have confidence in their ability to fulfill their roles. Counselors may feel unprepared to handle sensitive or difficult topics such as rape, especially if they have never experienced it or due to lack of training. Depending on the severity of the problem discussed, counselors may lose confidence in their ability to help the client solve the problem. Some clients may not feel like they had enough exposure to certain populations (e.g., some may feel more confident in counseling males than females). One counselor (A18/A11) felt that their client load affected their confidence in providing counseling; it seems like some clients were not attending counseling sessions and the counselor needs more resources. Another counselor though the training did not prepare them adequately for their role as counselors which can impact their confidence in their ability to implement the intervention.
- **Emergent Implementation Strategies:** More training, more experience with clients

**Facilitator # 2:** Counselors believe that implementation by peer counselors is generally acceptable and appropriate since they are similar age as their clients (this is contrary to Barrier # 1 regarding clients not trusting the counselors, at least initially).

- **CFIR Domain:** Characteristics of Individuals
- **CFIR Constructs:** Knowledge and Beliefs, Other Personal Attributes
- **Emergent Codes:** Facilitators, Client preferences and attitudes, Cultural factors related to age, Peer status
- **Summary:** Counselors found that their clients prefer communicating with their peers, or even “near peers” rather than older adults. Older adults lack an understanding of youth culture and therefore clients may not be able to be themselves if working with an older counselor. Due to social taboos, clients may not feel comfortable talking to older adults about their personal issues such as sex, relationships, and drug use. And older adults may not understand youth culture or “street language.” The counselors’ status as peers (or near-peers) is acceptable and appropriate because it makes them relatable to their clients. Peer relationships facilitate conversations about hard topics such as sex and cyber bullying. Age is a factor in terms of how well counselors will be able to relate to their clients. Due to their similar age category, counselors understand youth culture, lingo, and social media use. There also may be a sense of superiority when it comes to older adults, but as peers, counselors operate on the same level as their clients, and counselors try their best to communicate with their clients like friends and peers.

**Facilitator # 3:** Counselors are personally invested in Friendship Bench because they understand the benefits of their participation among themselves and for the clients they serve.

- **CFIR Domain:** Characteristics of Individuals (or Intervention Characteristics)
- **CFIR Constructs:** Knowledge and Beliefs, Other Personal Attributes (or Intervention Source, Evidence Strength and Quality, Relative Advantage)
- **Emergent Codes:** Facilitators, Counselor perceptions and motivations, Personal growth and satisfaction, Cultural factors related to age
- **Summary:** Counselors are personally motivated to participate in Friendship Bench. Positive counselor perceptions about Friendship Bench contributes to their intrinsic motivations to participate in the intervention. Their value and sense of self-fulfillment placed on the intervention is what motivates them to remain Friendship Bench. They understand the value of the intervention for their clients. Despite low compensation, counselors still found their participation in the intervention valuable. Some counselors see the positive impact that the intervention has on young people and see the personal progress in their clients, which may serve as a motivation for them to continue in their role as counselors. Many counselors found personal value in participating in Friendship Bench for themselves and for their clients. Counselors’ perceived value of the intervention may determine how motivated they are to participate in Friendship Bench. Friendship Bench served as a confidence booster for many counselors and gave counselors the ability to help their family and friends. Some counselors experienced positive changes in their character and wellness.

**II. Intervention Characteristics**

**Facilitator # 4:** Counselors believe that the intervention is working (this is related to Facilitator # 3).

- **CFIR Domain:** Intervention Characteristic
- **CFIR Constructs:** Evidence Strength and Quality, Relative Advantage
- **Emergent Codes:** Facilitators, Counselor perceptions and motivations
- **Summary:** The counselors acknowledge the impact that Friendship Bench is having on their clients’ lives with anecdotal stories of their client’s experiences (Evidence Strength and Quality) such as the clients are feeling confident in solving a problem on their own (i.e., confidence boosters). Counselors expressed that the peer-based counseling is even better than the traditional counseling provided by older adults (Relative Advantage).

**Barrier # 5:** Some counselors thought that the training covered to much information in a short period of time.

- **CFIR Domain:** Intervention Characteristic
- **CFIR Constructs:** Complexity
- **Emergent Codes:** Barriers, Training
- **Summary:** Some counselors expressed that the training sessions covered too much information in a short period of time. Counselors touched on concerns about retaining the information that they learned during the training, especially during periods when they are not seeing any clients (this was addressed in Barrier # 12). However, counselors found the training content to be helpful and necessary for helping them perform their jobs, but also expressed the additional training ~~needs~~ that they needed.
- **Implementation strategy:** Extend the length of the training

**III. Outer Setting**

**Barrier # 6:** The location of the intervention is not ideal for all clients and counselors.

- **CFIR Domain:** Outer Setting
- **CFIR Constructs:** Patient Needs and Resources
- **Emergent Codes:** Barriers, Client preferences and attitudes, Location of counseling sessions
- **Summary:** The location of the counseling sessions is not ideal for clients who live far away from Baylor, have disabilities, and can only attend during school hours. The location may also not be ideal for clients with parents who disapprove of the intervention. COVID-19 fears and protocols can also limit participation in counseling sessions. One counselor expressed their concern about COVID-19 exposure during attendance to the debriefing sessions. Some clients may not prefer the Baylor as a location since due to fears that their sessions will be shared with doctors (related to Barrier #1).
- **Emergent Implementation strategies:** Satellite locations and implementation in schools. Some counselors recommended having the sessions in school settings since the location of the sessions may not be ideal for clients who do not have parental approval to participate in the sessions and to avoid interfering with academic learning (e.g., have sessions during school lunch hours).

**Barrier # 7:** The duration and frequency of the counseling sessions may not be enough for clients with complex issues/needs.

- **CFIR Domain:** Outer Setting
- **CFIR Constructs:** Patient Needs and Resources
- **Related Emergent Codes:** Barriers, Duration and frequency of sessions
- **Summary:** The duration or frequency of the counseling sessions were not enough for some clients. Counselors mentioned that some issues take more or less time to work through.
- **Emergent Implementation Strategies:** Extend session length and frequency for clients who need it.

**Barrier # 8:** Societal views and cultural norms regarding counseling in Botswana may hinder client participation (related to Barrier # 2 and Facilitator # 2)

- **CFIR Domain:** Outer Setting
- **CFIR Constructs:** None
- **Emergent Codes:** Barriers, Duration and frequency of sessions, Societal norms and views
- **Summary:** Parents may not approve of clients receiving counseling as it may be viewed as a social taboo. Older people may not think that young people need counseling and may believe that young people do not have anxiety, depression, or substance abuse problems. Conversations about how clients may not be able to be themselves if working with an older counselor. This may also refer to how adolescents deal with stress (e.g., substance abuse).
- **Emergent Implementation Strategies:** Education about the importance of counseling and Friendship Bench in the community

**Barrier # 9:** Clients may have competing needs which may prevent participation in the intervention (not salient, only one counselor alluded to this)

- **CFIR Domain:** Outer Setting
- **CFIR Constructs:** Patient Needs and Resources
- **Emergent Codes:** Barriers, Competing needs
- **Summary:** Some clients may have competing needs that may prevent them from attending counseling sessions such as the need for money.

**IV. Inner Setting**

**Barrier # 10:** Counselors do not feel valued enough for their work

- **CFIR Domain:** Inner Setting
- **CFIR Constructs:** Learning Climate, Culture
- **Emergent Codes:** Barriers, Compensation and travel expenses, Counselor perceptions and motivations
- **Summary:** Some clients do not feel valued enough for their work due to low compensation whereby others did feel valued enough by their peers or by leaders.
- **Implementation strategies:** Options for tenure, higher compensation (can improve motivation)

**Barrier # 11:** Counselors do not have enough money for transportation to the intervention site.

- **CFIR Domain:** Inner Setting
- **CFIR Constructs:** Available Resources
- **Emergent Codes:** Barriers, Compensation and travel expenses
- **Summary:** A counselor raised a concern that they were not getting enough money for transport, and the combi prices went up.
- **Implementation strategies:** Higher compensation (can improve motivation)

**Barrier # 12:** Some counselors have conflicts or lack trust among their peers.

- **CFIR Domain:** Inner Setting
- **CFIR Constructs:** Culture, Networks and Communications
- **Emergent Codes:** Barriers, Coworker trust, Coworker conflict
- **Summary:** Some counselors are hesitant to share information during debriefing or support sessions out of fear that a colleague will disclose the information outside of the sessions and there may be conflict between counselors which can hinder performance or implementation of the intervention. One counselor stated that they do not get along with one of the other counselor. A few counselors mentioned that there was a “snitch” who disclosed information shared during the briefing sessions.
- **Implementation strategies:** Mediation among leadership staff

**Facilitator # 5:** Counselors feel supported by leadership.

- **CFIR Domain:** Inner Setting
- **CFIR Constructs:** Leadership Engagement, Networks and Communications
- **Emergent Codes:** Facilitators, Leadership and staff support
- **Summary***:* The extent to which counselors feel supported by leadership, overseers of the intervention, and staff psychologists. This also includes discussions about how counselors are emotionally supported during debrief and support sessions and how counselors expressed the emotional support they needed (and what they found helpful).

**V. Process**

**Barrier # 13:** Counselors believe that they were not getting enough clients (a part of it due to COVID).

- **CFIR Domain:** Process
- **CFIR Constructs:** Reflecting and Evaluating, Executing
- **Emergent Codes:** Barriers, Recruitment and client load
- **Summary:** Some counselors thought that clients were not being screened fast enough which was preventing them from seeing more clients. Counselors wished to have more clients than what they were getting. One counselor expressed the concern that if they were not getting enough clients, they will eventually forget the information they learned during the training.
- **Emergent Implementation Strategies:** Have the counselors screen and recruit clients. . Some counselors suggested that they do the screenings to improve recruitment of clients and raise awareness about the intervention in the community.

**Barrier # 14:** Counselors have issues with client attendance and scheduling procedures.

- **CFIR Domain:** Process
- **CFIR Constructs:** Reflecting and Evaluating, Executing
- **Emergent Codes:** Barriers, Scheduling procedures
- **Summary:** Some clients were not attending their counseling appointments on time or were not showing up. As a result, counselors either wasted travel time and money to the counseling location and had to return later when the client arrived.
- **Implementation strategy:** Counselors were in favor of the block system which allowed them to reserve a few hours each week for clients. This block system seemed to address the issue of clients showing up late or not showing up at all. Some counselors suggested strategies for improving scheduling and attendance such as hiring full-time workers.

**Facilitator # 6:** The debriefing and counseling sessions were helping the counselors deal with issues they face with their clients, and the counseling was helpful for meeting their own psychosocial needs.

- **CFIR Domain:** Process
- **CFIR Constructs:** Reflecting and Evaluating, Executing
- **Emergent Codes:** Barriers, Scheduling procedures
- **Summary:** If the counselors feel they have adequate support, this can motivate them to stay involved in the intervention. Some counselors have expressed that the debriefing sessions were not long enough, or that they wish that meals were provided during these sessions. However, counselors generally found these sessions helpful and thought that the debrief and support sessions prepared them for counseling sessions with their clients.

**Conclusions**

- Counselors also serving as advocates to address the needs of their clients
- Meeting patient needs, patient-centeredness (e.g., offering sessions virtually, education around COVID-19)
- Some barriers/facilitators can fit into more than one CFIR domain
- CFIR constructs are not all-inclusive: need to expand or tailor domains and constructs in CFIR to fit context

**Theme Development Meeting**

*
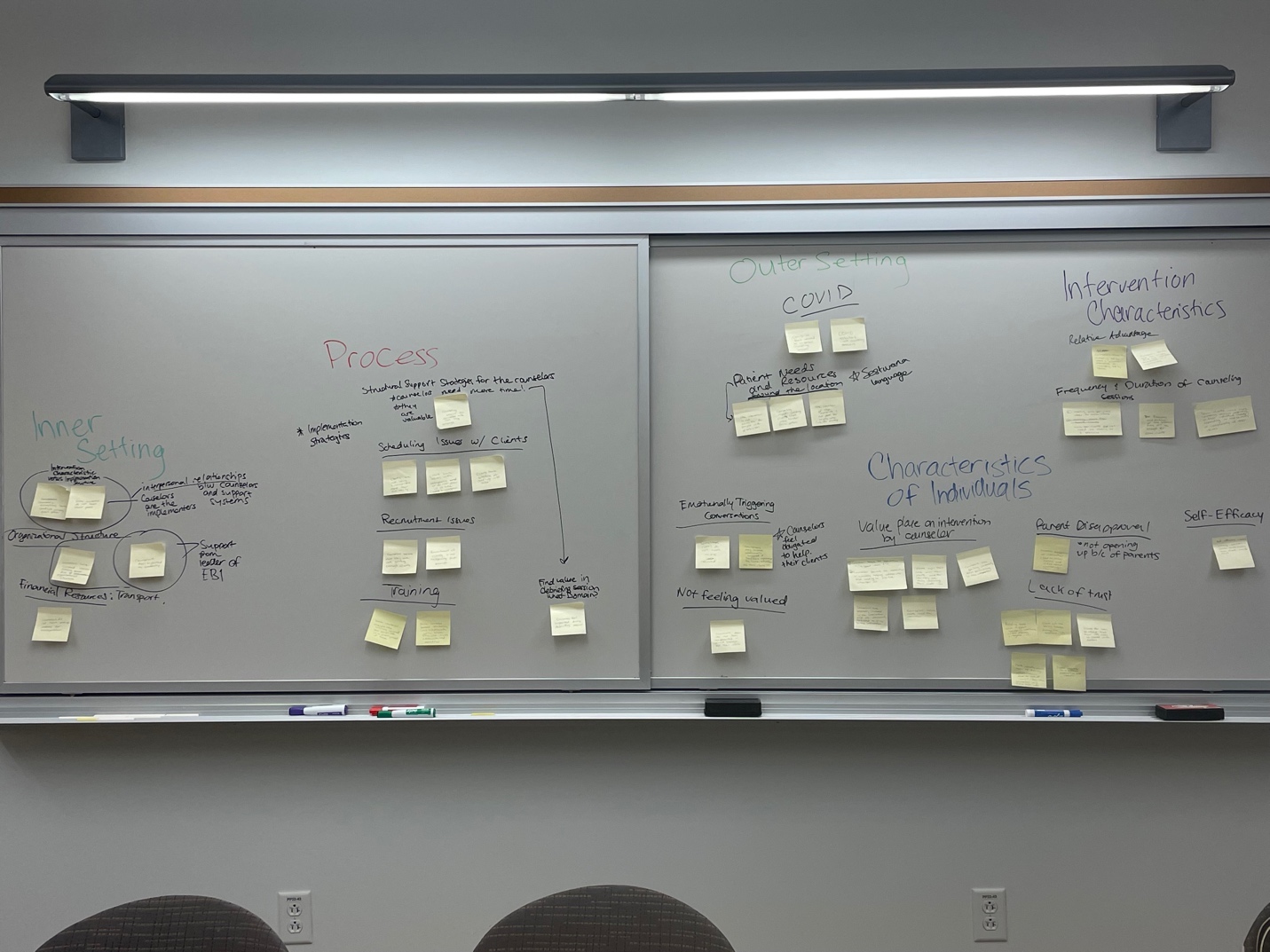
*
